# Isolation of Progenitors that Exhibit Myogenic/Osteogenic Bipotency In Vitro by Fluorescence-Activated Cell Sorting from Human Fetal Muscle

**DOI:** 10.1016/j.stemcr.2013.12.006

**Published:** 2014-01-14

**Authors:** Alessandra Castiglioni, Simone Hettmer, Matthew D. Lynes, Tata Nageswara Rao, Daria Tchessalova, Indranil Sinha, Bernard T. Lee, Yu-Hua Tseng, Amy J. Wagers

**Affiliations:** 1 Howard Hughes Medical Institute, Department of Stem Cell and Regenerative Biology, Harvard University, Harvard Stem Cell Institute, Cambridge, MA 02138, USA; 2 Joslin Diabetes Center and the Paul F. Glenn Laboratories for the Biological Mechanisms of Aging, Harvard Medical School, Boston, MA 02115, USA; 3 Vita-Salute San Raffaele University, Milan 20132, Italy; 4 Department of Pediatric Oncology, Dana Farber Cancer Institute and Division of Pediatric Hematology/Oncology, Children’s Hospital, Boston, MA 02115, USA; 5 Division of Plastic Surgery, Brigham and Women’s Hospital, Boston, MA 02115, USA; 6 Division of Plastic and Reconstructive Surgery, Department of Surgery, Beth Israel Deaconess Medical Center, Boston, MA 02215, USA

## Abstract

Fluorescence-activated cell sorting (FACS) strategies to purify distinct cell types from the pool of fetal human myofiber-associated (hMFA) cells were developed. We demonstrate that cells expressing the satellite cell marker PAX7 are highly enriched within the subset of CD45^−^CD11b^−^GlyA^−^CD31^−^CD34^−^CD56^int^ITGA7^hi^ hMFA cells. These CD45^−^CD11b^−^GlyA^−^CD31^−^CD34^−^CD56^int^ITGA7^hi^ cells lack adipogenic capacity but exhibit robust, bipotent myogenic and osteogenic activity in vitro and engraft myofibers when transplanted into mouse muscle. In contrast, CD45^−^CD11b^−^GlyA^−^CD31^−^CD34^+^ fetal hMFA cells represent stromal constituents of muscle that do not express PAX7, lack myogenic function, and exhibit adipogenic and osteogenic capacity in vitro. Adult muscle likewise contains PAX7^+^ CD45^−^CD11b^−^GlyA^−^CD31^−^CD34^−^CD56^int^ITGA7^hi^ hMFA cells with in vitro myogenic and osteogenic activity, although these cells are present at lower frequency in comparison to their fetal counterparts. The ability to directly isolate functionally distinct progenitor cells from human muscle will enable novel insights into muscle lineage specification and homeostasis.

## Highlights

• CD45^−^CD11b^−^GlyA^−^CD31^−^CD34^−^CD56^int^ITGA7^hi^ human fetal muscle cells are PAX7^+^
• CD34^−^CD56^int^ITGA7^hi^ fetal cells have bipotent myogenic/osteogenic activity in vitro
• PAX7^+^ CD34^−^CD56^int^ITGA7^hi^ cells are fewer in number in adult versus fetal muscle

## Teaser

This work by Castiglioni et al. establishes the presence of functionally distinct cell populations within the pool of myofiber-associated cells in human fetal and adult skeletal muscle and reports effective methods for the direct isolation by fluorescence-activated cell sorting of a cell population in fetal muscle with bipotent in vitro myogenic/osteogenic activity.

## Introduction

In mice, combinatorial surface marker analysis has been useful in enabling direct discrimination and prospective isolation of phenotypically and functionally distinct cells from skeletal muscle using fluorescence-activated cell sorting (FACS) (Cerletti et al., 2008, Kuang et al., 2007, Sacco et al., 2008, Sherwood et al., 2004, Tanaka et al., 2009). FACS has been used to purify PAX7-expressing mouse satellite cells, which exhibit self-renewal and myogenic differentiation capacities consistent with muscle stem cells (Cerletti et al., 2008, Fukada et al., 2004, Kuang et al., 2007, Montarras et al., 2005, Sacco et al., 2008, Sherwood et al., 2004, Tanaka et al., 2009). Prospective isolation of adult mouse satellite cells has also enabled studies that distinguished their myogenic differentiation potential from adipogenic/fibrogenic activities in muscle (Joe et al., 2010), revealed their contributions to muscle pathologies (Cerletti et al., 2008, Chakkalakal et al., 2012, Conboy et al., 2003, Sacco et al., 2008), and provided proof in principle that they may be useful in cell therapy approaches (Cerletti et al., 2008, Cerletti et al., 2012, Sacco et al., 2008). A similar cell-sorting approach recently allowed purification of fibroadipogenic precursors from mouse muscle and showed that these cells lack myogenic capacity (Joe et al., 2010, Uezumi et al., 2010). Together with endothelial and infiltrating immune cells, these fibroadipogenic precursors constitute the muscle stroma and play a critical role in regulating the early stages of muscle repair after damage (Wang and Rudnicki, 2012). However, in order to translate these findings to human muscle and apply them for regenerative medicine, it is essential to develop analogous strategies for prospective identification and isolation of human myogenic and adipogenic precursors.

Lecourt et al. previously showed by immunofluorescence (IF) staining that cells in the satellite cell position in adult human muscle lack CD34 (Lecourt et al., 2010). Pisani et al. subsequently demonstrated that myogenic activity could be enriched in human adult muscle cells by magnetic depletion of CD34^+^ cells (Pisani et al., 2010b). However, as described here, negative selection for CD34 achieves only partial purification of myogenic progenitors from human fetal muscle. To establish more specific sorting strategies capable of purifying human PAX7-positive cells, we undertook a systematic study of surface markers that distinguish phenotypically and functionally distinct cells in human fetal muscle. These efforts identified a combination of seven surface markers that reliably discriminate a purified population of PAX7-expressing CD45^−^CD11b^−^GlyA^−^CD31^−^CD34^−^CD56^int^ITGA7^hi^ human myofiber-associated (hMFA) cells (hereafter referred to as CD34^−^CD56^int^ITGA7^hi^ cells) from infiltrating blood cells and muscle-resident adipogenic precursors, allowing direct isolation of each of these populations by FACS. Consistent with studies in the mouse, human PAX7-expressing CD34^−^CD56^int^ITGA7^hi^ cells are robustly myogenic and lack adipogenic potential. PAX7-expressing CD34^−^CD56^int^ITGA7^hi^ cells with myogenic activity in vitro are also present in adult muscle, but at lower frequency than in fetal tissue. Clonal analysis in vitro further revealed a surprising bipotency of human fetal PAX7-expressing CD34^−^CD56^int^ITGA7^hi^ cells, which exhibited both myogenic and osteogenic potential. In contrast, CD45^−^CD11b^−^GlyA^−^CD31^−^CD34^+^ fetal hMFA cells (abbreviated CD34^+^ cells), which exhibited potent adipogenic and osteogenic activity, lack PAX7 and show no myogenic potential. Taken together, these studies report efficient methods for the direct isolation of highly enriched human fetal bipotent myogenic/osteogenic and adipogenic progenitors. These protocols provide tools for uncovering the cellular mechanisms and environmental interactions that sustain human skeletal muscle.

## Results

### Human Fetal Skeletal Muscle Contains Multiple, Distinct Cell Populations

**Figure 1:**
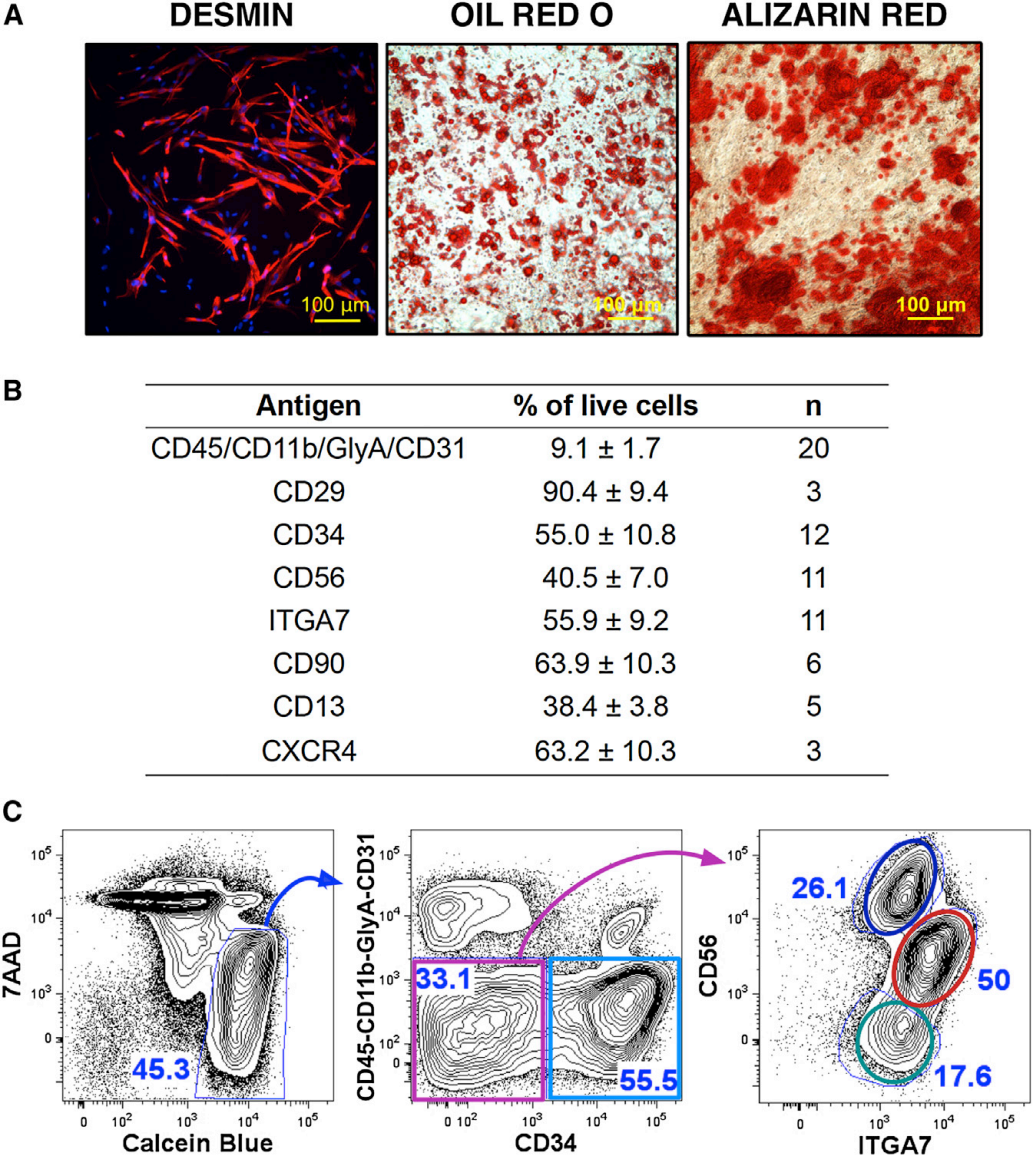
Cellular Heterogeneity in Human Fetal Muscle (A) hMFA cell differentiation under myogenic, adipogenic, and osteogenic conditions. Unfractionated hMFA cells formed DESMIN^+^, multinucleated myocytes (left panel), oil red O^+^ adipocytes (central panel), and Alizarin red^+^ calcium deposits (right panel) under myogenic (left), adipogenic (middle), or osteogenic (right) conditions. (B) Surface antigen expression within live (Pi^−^Ca^+^ or 7AAD^−^Ca^+^) hMFA cells (mean ± SD based on 3–20 biologically distinct samples). (C) Discrimination of phenotypically distinct hMFA cells. Viable hMFA cells were 7AAD^−^ and Calcein blue^+^ (left). Expression of CD34 discriminated two populations within CD45^−^CD11b^−^GlyA^−^CD31^−^ cells (middle). Among CD34^−/low^ cells (pink gate), ITGA7 and CD56 identify three additional populations: CD34^−^CD56^hi^ITGA7^low^ (blue gate), CD34^−^CD56^int^ITGA7^hi^ (red gate), and CD34^−/low^CD56^−^ITGA7^low^ (green gate) cells (right). See also Figure S1.

To evaluate phenotypic and functional heterogeneity among fetal hMFA cells, we adapted previously established protocols for mouse myofiber-associated cell isolation (Conboy et al., 2003, Sherwood et al., 2004) to liberate the mononuclear cell fraction from human fetal muscle (Ehrhardt et al., 2007, Tanaka et al., 1995). Plating hMFA cells under myogenic, adipogenic, or osteogenic conditions in vitro revealed significant functional heterogeneity. Under myogenic conditions, hMFA cells formed DESMIN-expressing multinucleated myotubes. Under adipogenic conditions, hMFA cells differentiated into oil red O (ORO)-positive, lipid droplet-containing adipocytes. Under osteogenic conditions, hMFA cells produced Alizarin-red (AR)-positive calcium deposits consistent with osteogenic differentiation (Figure 1A).

We next evaluated phenotypic heterogeneity within the hMFA pool using cell surface marker staining and FACS. Existing literature was surveyed to identify candidate antigenic markers that might discriminate live myogenic from nonmyogenic cells (Fukada et al., 2004, Kuang et al., 2007, Lecourt et al., 2010, Pisani et al., 2010a, Pisani et al., 2010b, Sacco et al., 2008, Sherwood et al., 2004). PAX7, the canonical marker of muscle satellite cells in mouse and human postnatal muscle (Bosnakovski et al., 2008, Seale et al., 2000), is inappropriate for such an approach because as a nuclear protein, antibody staining requires cell fixation/permeabilization. Flow cytometric analysis of hMFA cells revealed differential expression of 11 candidate cell surface markers (CD45, CD11b, glycophorin A [GlyA], β1 integrin, CD34, CD56, ITGA7, CD90, CD13, and CXCR4; Figure 1B and Figure S1A available online). A total of 9.1% ± 1.7% (mean ± SD) of fetal hMFA cells expressed hematopoietic lineage markers (CD45, CD11b, and GlyA) and CD31, an endothelial marker (Figure 1B) (Andukuri et al., 2013). Expression of CD34, CD56, and ITGA7 was detected in 55% ± 10.8%, 40.5% ± 7.0%, and 55.9% ± 9.2% of cells, respectively (mean ± SD; Figures 1B and S1A–S1C). Other markers analyzed included CD29, CD90, CD13, and CXCR4, which were expressed by 90.4% ± 9.4% (CD29), 63.9% ± 10.3% (CD90), 38.4% ± 3.8% (CD13), and 63.2% ± 10.3% (CXCR4) of cells, respectively (mean ± SD; Figures 1B and S1D–S1F). These analyses confirmed heterogeneity of cell surface marker expression by fetal hMFA cells. We therefore sought to exploit this heterogeneity to fractionate fetal hMFA subsets with distinct differentiation potentials.

### CD45^−^CD11b^−^GlyA^−^CD31^−^CD34^−^CD56^int^ITGA7^hi^ Fetal hMFA Cells Are Enriched for PAX7-Expressing Cells

**Figure 2:**
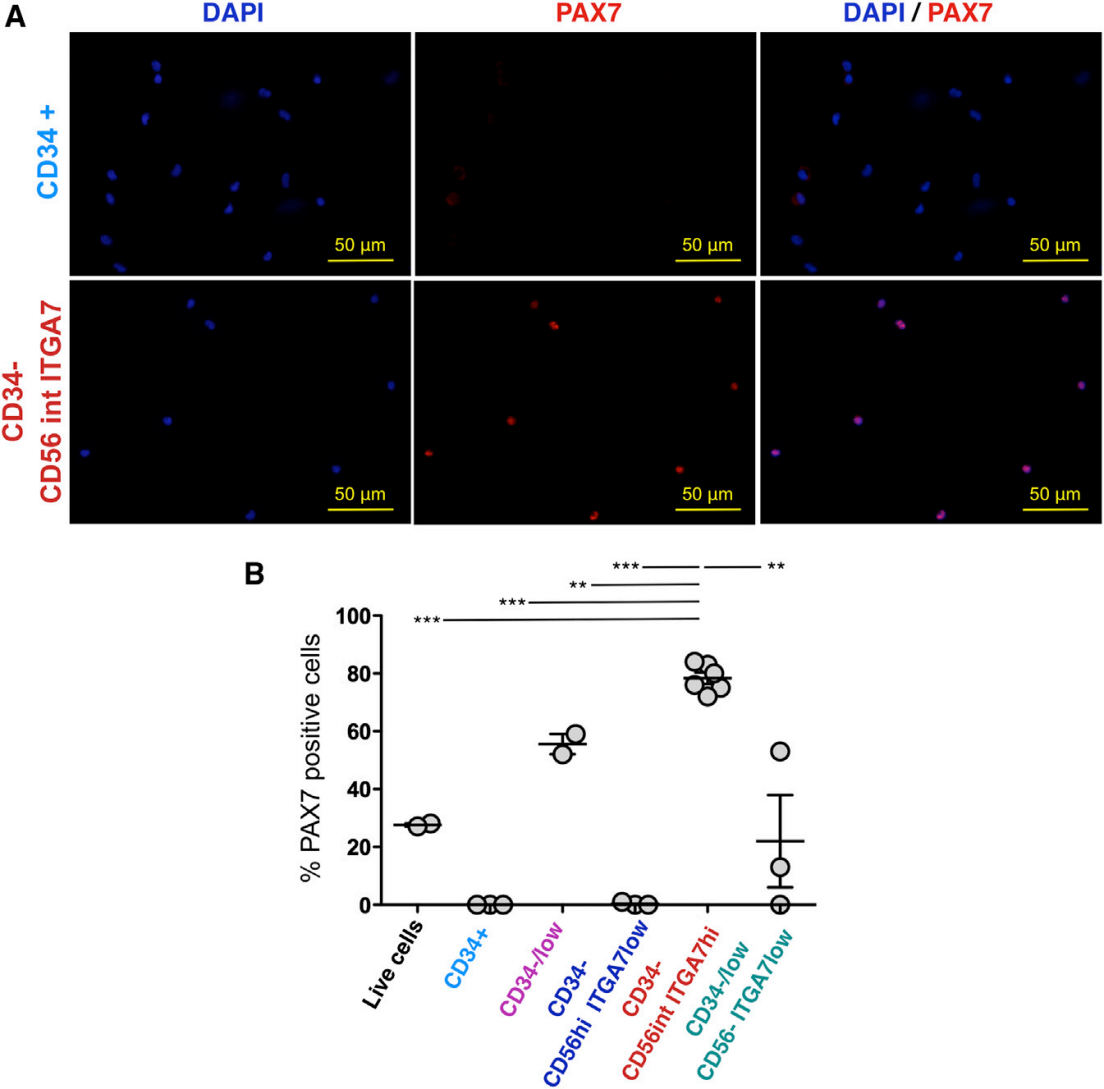
PAX7 Enrichment in CD34^−^CD56^int^ITGA7^hi^ Fetal hMFA Cells (A and B) PAX7 expression in fetal hMFA cell subsets was (A) determined by IF of freshly sorted cells and (B) quantified as the percentage of PAX7^+^ cells among all DAPI^+^ cells (mean ± SD; two to six biological replicates). CD34^−^CD56^int^ITGA7^hi^ cells are highly enriched for PAX7 (mean ± SD, 78.3% ± 5%). Statistical significance was evaluated by unpaired, two-tailed t test (^∗∗^p < 0.001; ^∗∗∗^p < 0.0001). See also Figure S2.

Satellite cells are canonically recognized by expression of the paired box transcription factor PAX7, which controls transcription of myogenic genes such as *MyoD* and *Myf5* (McKinnell et al., 2008) and is present in satellite cells as well as muscle progenitors in postnatal muscle tissue. A total of 27.5% ± 1% (mean ± SD) of fetal hMFA cells expressed PAX7 by IF analysis (Figures 2B and S2). To assess PAX7 expression by IF in fetal hMFA cell subsets, cells were isolated by FACS after combinatorial staining for differentially expressed cell surface markers (Figure 1C). Exclusion of cells expressing CD45, CD11b, GlyA, and CD31 (which mark hematopoietic and endothelial lineage cells) and selection of CD34^−/low^ cells identified a population enriched for PAX7-expressing cells (55% ± 5% PAX7^+^, mean ± SD; Figure 2B). These CD45^−^CD11b^−^GlyA^−^CD31^−^CD34^−/low^ cells are hereafter designated “CD34^−/low^ cells” (marked in pink in Figure 1C). None of the CD45^−^CD11b^−^GlyA^−^CD3^−^CD34^+^ cells (hereafter designated “CD34^+^ cells” and marked in cyan in Figure 1C) expressed PAX7 (Figure 2). Thus, CD34^−/low^ hMFA cells from fetal muscle are selectively enriched for PAX7-expressing cells.

To further enrich PAX7^+^ cells within the CD34^−/low^ hMFA cell subset, we evaluated expression of additional surface markers (Figure 1B). Differential expression of CD90, CD13, and CD29 did not discriminate candidate populations within the CD34^−/low^ hMFA cell pool, as CD90 and CD13 expression was enriched in the CD34^+^ cell subset and CD29 was uniformly expressed by CD34^−/low^ cells (data not shown). In contrast, differential expression of CD56 and ITGA7 distinguished three populations within CD34^−/low^ fetal hMFA cells: CD34^−^CD56^hi^ITGA7^low^ (marked in blue in Figure 1C), CD34^−^CD56^int^ITGA7^hi^ (marked in red in Figure 1C), and CD34^−/low^CD56^−^ITGA7^low^ (marked in green in Figure 1C). In addition to differences in CD56 and ITGA7 expression, variable low-level expression of CD34 was noted in CD34^−/low^CD56^−^ITGA7^low^ hMFA cells, compared to absent CD34 expression in CD34^−^CD56^int^ITGA7^hi^ and CD34^−^CD56^hi^ITGA7^low^ cells (Figure S1H). PAX7 IF showed clear enrichment of PAX7+ cells (78.3% ± 5%, mean ± SD) in the CD34^−^CD56^int^ITGA7^hi^ subset (Figure 2; red gate in Figure 1C). In contrast, we detected no PAX7 expression in CD34^−^CD56^hi^ITGA7^low^ cells (Figures 2B and S2; blue gate in Figure 1C) and variable PAX7 expression in CD34^−/low^CD56^−^ ITGA7^low^ cells (33.0% ± 28%, mean ± SD; Figures 2B and S2; green gate in Figure 1C).

All cell populations were sorted twice to maximize purity. The purity of double-sorted CD34^+^, CD34^−/low^, CD34^−^CD56^hi^ITGA7^low^, and CD34^−^CD56^int^ITGA7^hi^ cells was consistently >99% upon reanalysis (Figure S3); however, reanalysis of sorted CD34^−/low^CD56^−^ITGA7^low^ cells showed variable purities of 68.1% ± 26% (mean ± SD; Figure S3). Variable contamination with CD34^−^CD56^int^ITGA7^hi^ cells could explain the variable levels of PAX7 expression detected in the sorted CD34^−/low^CD56^−^ITGA7^low^ cells. In contrast, PAX7 enrichment in CD34^−^CD56^int^ITGA7^hi^ cells and absent PAX7 expression in CD34^−^CD56^hi^ITGA7^low^ was highly reproducible (n = 6 distinct donors for CD34^−^CD56^int^ITGA7^hi^ cell analysis and n = 3 donors for CD34^−^CD56^hi^ITGA7^low^ cell analysis; Figure 2B). Thus, the canonical satellite cell marker PAX7 is selectively enriched in CD34^−^CD56^int^ITGA7^hi^ fetal hMFA cells, suggesting that human myogenic progenitors may be contained in this population.

### CD34^−^CD56^int^ITGA7^hi^ Fetal hMFA Cells Exhibit Myogenic and Osteogenic Activity In Vitro but Lack Adipogenic Differentiation Potential

**Figure 3:**
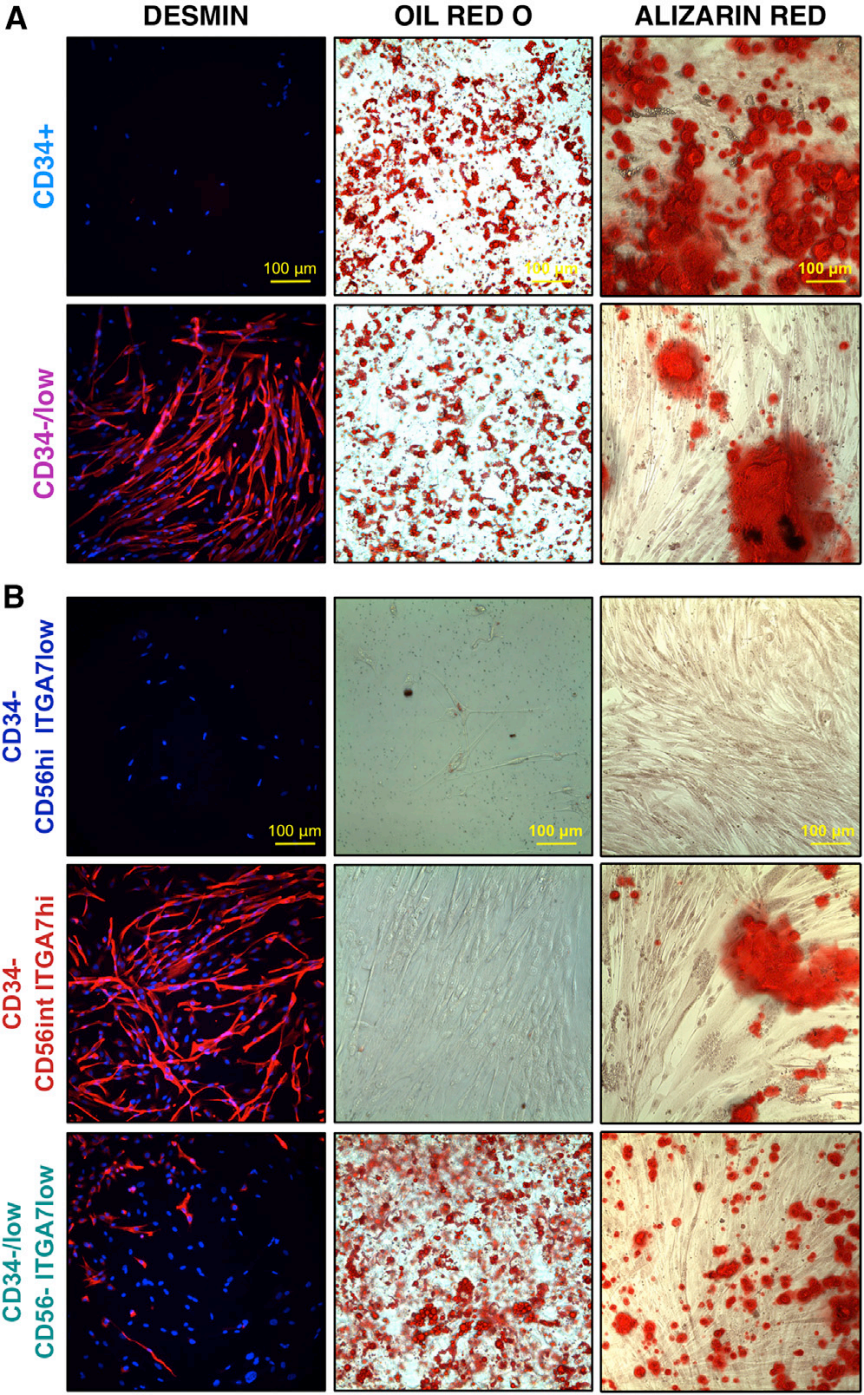
Differentiation of Fetal hMFA Cell Subsets under Myogenic, Adipogenic, and Osteogenic Conditions (A and B) hMFA cells were stained for DESMIN (red) and nuclei marked by DAPI (blue) after myogenic culture (left panels), with oil red O to mark lipid droplets after adipogenic culture (middle panels), and with Alizarin red to mark calcium deposits after osteogenic culture (right panels). (A) CD34^+^ cells lack myogenic activity but are adipogenic and osteogenic in vitro, while fetal CD34^−/low^ cells contain myogenic, adipogenic, and osteogenic activity in vitro. (B) CD34^−^CD56^hi^ITGA7^low^ cells lack myogenic, adipogenic, and osteogenic capacity in vitro. CD34^−^CD56^int^ITGA7^hi^ cells exhibit efficient myogenic activity, lack adipogenic potential, and contain osteogenic activity in vitro. CD34^−/low^CD56^−^ITGA7^low^ cells show limited myogenic activity but are adipogenic and osteogenic in vitro. Differentiation assays were performed in three biological replicates for each population. See also Figure S4.

We next evaluated the lineage potential of the fetal hMFA cell subsets identified above using in vitro differentiation assays. Under myogenic conditions (Figure 3, left panels), CD34^−/low^ and CD34^−^CD56^int^ITGA7^hi^ cells exhibited efficient myogenic differentiation, as evidenced by large numbers of DESMIN^+^ multinucleated myotubes. In contrast, CD34^−/low^CD56^−^ITGA7^low^ cells showed minimal myogenic capacity. CD34^+^ and CD34^−^CD56^hi^ITGA7^low^ cells, both of which lack PAX7 expression (Figure 2), exhibited no myogenic activity. Under adipogenic conditions (Figure 3, central panels), both CD34^+^ and CD34^−/low^ cells formed adipocytes (containing ORO-staining lipid droplets). Within the CD34^−/low^ subset, only CD34^−/low^CD56^−^ITGA7^low^ cells contained adipogenic activity, while CD34^−^CD56^int^ITGA7^hi^ and CD34^−^CD56^hi^ITGA7^low^ cells were uniformly nonadipogenic. These data suggest that CD34^−/low^CD56^−^ITGA7^low^ cells are responsible for the adipogenic differentiation potential present among CD34^−/low^ fetal hMFA cells. Finally, under osteogenic conditions (Figure 3, right panels), both CD34^+^ and CD34^−/low^ cells formed AR-staining calcium deposits, consistent with osteogenic differentiation. Myogenic CD34^−^CD56^int^ITGA7^hi^ and CD34^−/low^CD56^−^ITGA7^low^ cells also exhibited osteogenic activity in these in vitro assays (Figure 3B, right panels).

Differences in the myogenic and adipogenic differentiation capacity of fetal CD34^−^CD56^int^ITGA7^hi^ cells and CD34^+^ cells remained evident under all the culture conditions used. Specifically, myogenic CD34^−^CD56^int^ITGA7^hi^ cells still formed multinucleated myotubes under adipogenic (Figure S4A) and osteogenic (Figures 3B and S4B) conditions and never formed adipocytes (Figures S4A and S4B). In contrast, CD34^+^ cells formed adipocytes under adipogenic (Figures 3 and S4A) and osteogenic (Figure S4B) conditions and never formed myotubes (Figures 3 and S4A). Under myogenic conditions, CD34^+^ cells adopted fibroblastic morphology (data not shown).

**Figure 4:**
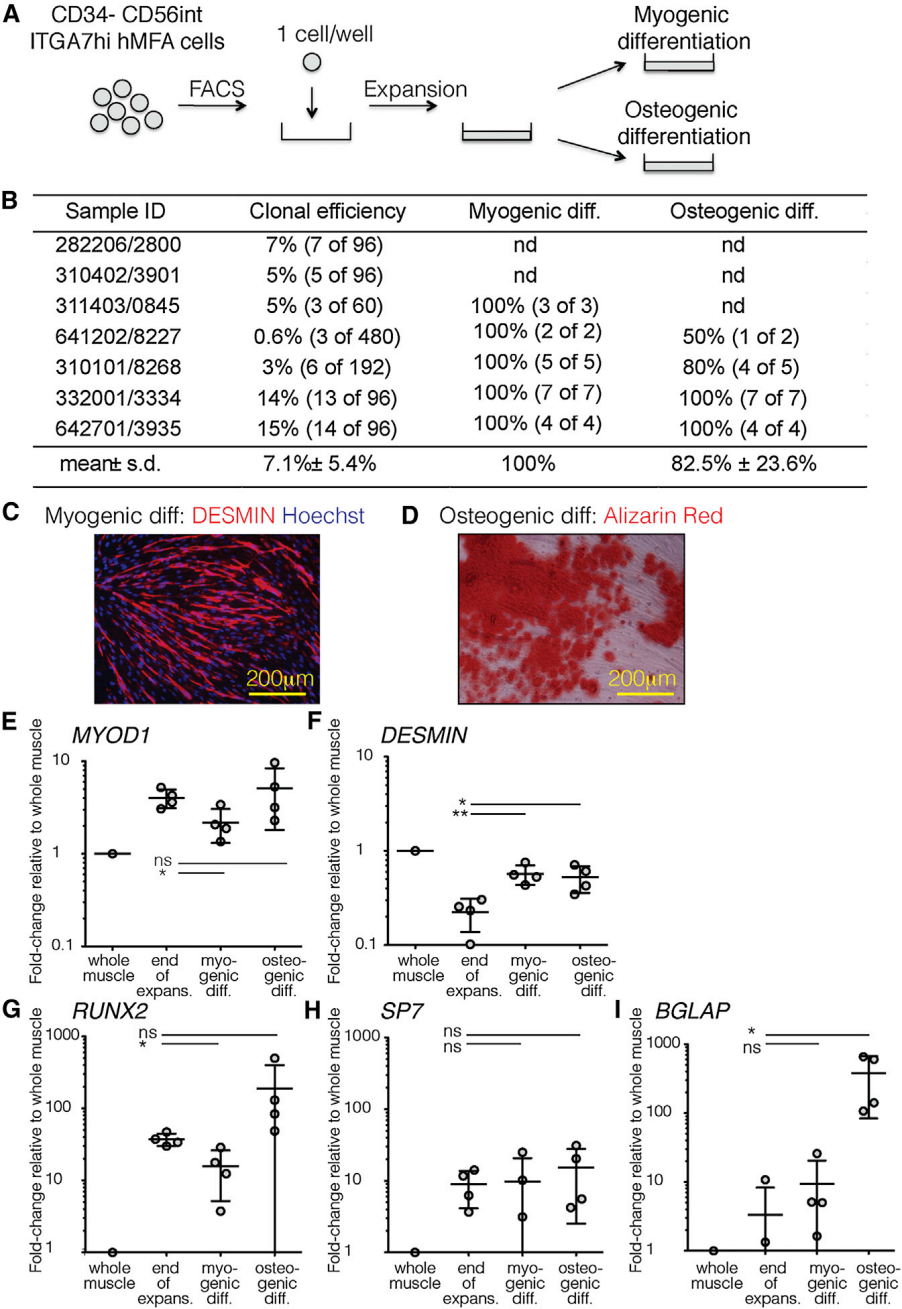
Bipotent Osteogenic/Myogenic Activity of CD34^−^CD56^int^ITGA7^hi^ Fetal hMFA Cells In Vitro (A) CD34^−^CD56^int^ITGA7^hi^ hMFA cells were sorted at 1 cell per well into 96-well plates. The clones were expanded and replated under myogenic or osteogenic conditions. (B) The colony-formation, myogenic, and osteogenic differentiation capacity of clones was evaluated. Myogenic differentiation was observed in 100% of clones and osteogenic differentiation in 83% (mean; range 50%–100%) of clones (isolated from four donors each). (C) Myogenic differentiation (diff) capacity was evaluated by IF for DESMIN (representative image of 1 of 18 clones). (D) Osteogenic capacity was evaluated by Alizarin red staining (representative image of 1 of 18 clones). (E–I) Myogenic (*MYOD* and *DESMIN*) and osteogenic (*RUNX2*, *SP7*, *BGLAP*) differentiation genes were evaluated by qRT-PCR at the end of expansion (expans.), myogenic, and osteogenic differentiation. Fold-change differences relative to whole human muscle were calculated for each gene and condition and confirm the bipotency of CD34^−^CD56^int^ITGA7^hi^ hMFA cells. This assay was replicated in four biologically independent experiments. Statistical significance was evaluated by unpaired, two-tailed t test (^∗^p < 0.05; ^∗∗^p < 0.001; ns, not significant). Data are mean ± SD. See also Figure S3.

In summary, these experiments reveal the following differences in the in vitro myogenic, adipogenic, and osteogenic potentials of discrete, prospectively isolatable fetal hMFA cell subsets: (1) PAX7-negative, CD34^−^CD56^hi^ITGA7^low^ cells lack myogenic, adipogenic, and osteogenic activity (Figures 2 and 3B); (2) PAX7-negative, CD34^+^ cells contain adipogenic and osteogenic activity but lack myogenic capacity (Figure 2 and Figure 3A); and (3) PAX7-positive, CD34^−^CD56^int^ITGA7^hi^ cells (Figure 2) contain myogenic and osteogenic capacity (Figure 4) but lack adipogenic potential (Figure 3B).

### Human Fetal CD34^−^CD56^int^ITGA7^hi^ Cells Exhibit Bipotent Myogenic/Osteogenic Differentiation Activity In Vitro

The ability of PAX7-positive, CD34^−^CD56^int^ITGA7^hi^ human fetal cells to efficiently undergo myogenic differentiation in vitro and form AR-positive calcium deposits indicative of osteogenic differentiation is consistent with previous reports of hMFA osteogenic differentiation (Hashimoto et al., 2008, Lecourt et al., 2010, Oishi et al., 2013). To determine whether this observation reflects true bipotency, we clone-sorted CD34^−^CD56^int^ITGA7^hi^ cells into 96-well plates (one cell per well) and expanded them for parallel analysis of myogenic and osteogenic activity in vitro (Figure 4A). The efficiency of CD34^−^CD56^int^ITGA7^hi^ fetal hMFA cell clonal expansion was variable and donor dependent, with an average seeding efficiency of 7.1% ± 5.4% (mean ± SD; Figure 4B). We succeeded in expanding 18 clones from four donors. After 14–22 days of expansion, these 18 clones were split and replated under myogenic or osteogenic differentiation conditions. Under myogenic conditions, 100% (Figure 4B) of clones differentiated into DESMIN-positive myotubes (Figure 4C; one representative clone shown). Under osteogenic conditions, 82.5% ± 23.6% (mean ± SD; Figure 4B) of clones formed AR-positive calcium deposits (Figure 4D; one representative clone shown). We conclude that the majority of fetal CD34^−^CD56^int^ITGA7^hi^ hMFA cells possess bipotent myogenic and osteogenic differentiation potential in these in vitro assays.

To further investigate the potential of fetal CD34^−^CD56^int^ITGA7^hi^ cells, we determined their expression of myogenic and osteogenic lineage genes at the end of clonal expansion and after myogenic or osteogenic differentiation (Figure 4A). All (four out of four) CD34^−^CD56^int^ITGA7^hi^ cell-derived clones analyzed by quantitative RT-PCR (qRT-PCR) expressed both myogenic (*MYOD* and *DESMIN*; Figures 4E and 4F) and osteogenic (*RUNX2*, *OSTERIX/SP7*, and *OSTEOCALCIN/BGLAP*; Figures 4G–4I) genes. Osteogenic lineage gene expression at the end of in vitro clonal expansion and after myogenic and osteogenic differentiation corroborated the osteogenic activity of human fetal CD34^−^CD56^int^ITGA7^hi^ cells. Expression of *BGLAP* increased significantly in cells that underwent osteogenic differentiation compared to proliferating clones; however, *BGLAP* did not increase in cells that underwent myogenic differentiation (Figure 4I). Increased expression of *RUNX2* and *SP7* in CD34^−^CD56^int^ITGA7^hi^ cells after osteogenic differentiation in vitro was variable between clones and did not reach statistical significance (Figures 4G and 4H). Importantly, at least some CD34^−^CD56^int^ITGA7^hi^ cells exposed to osteogenic differentiation conditions maintained myogenic activity as evidenced by an increase in *DESMIN* expression during osteogenic culture (Figures 4F) and the presence of multinucleated myotubes in these cultures (data not shown).

### Human Fetal CD34^−^CD56^int^ITGA7^hi^ Cells Engraft Myofibers when Transplanted into Mouse Skeletal Muscle

**Figure 5:**
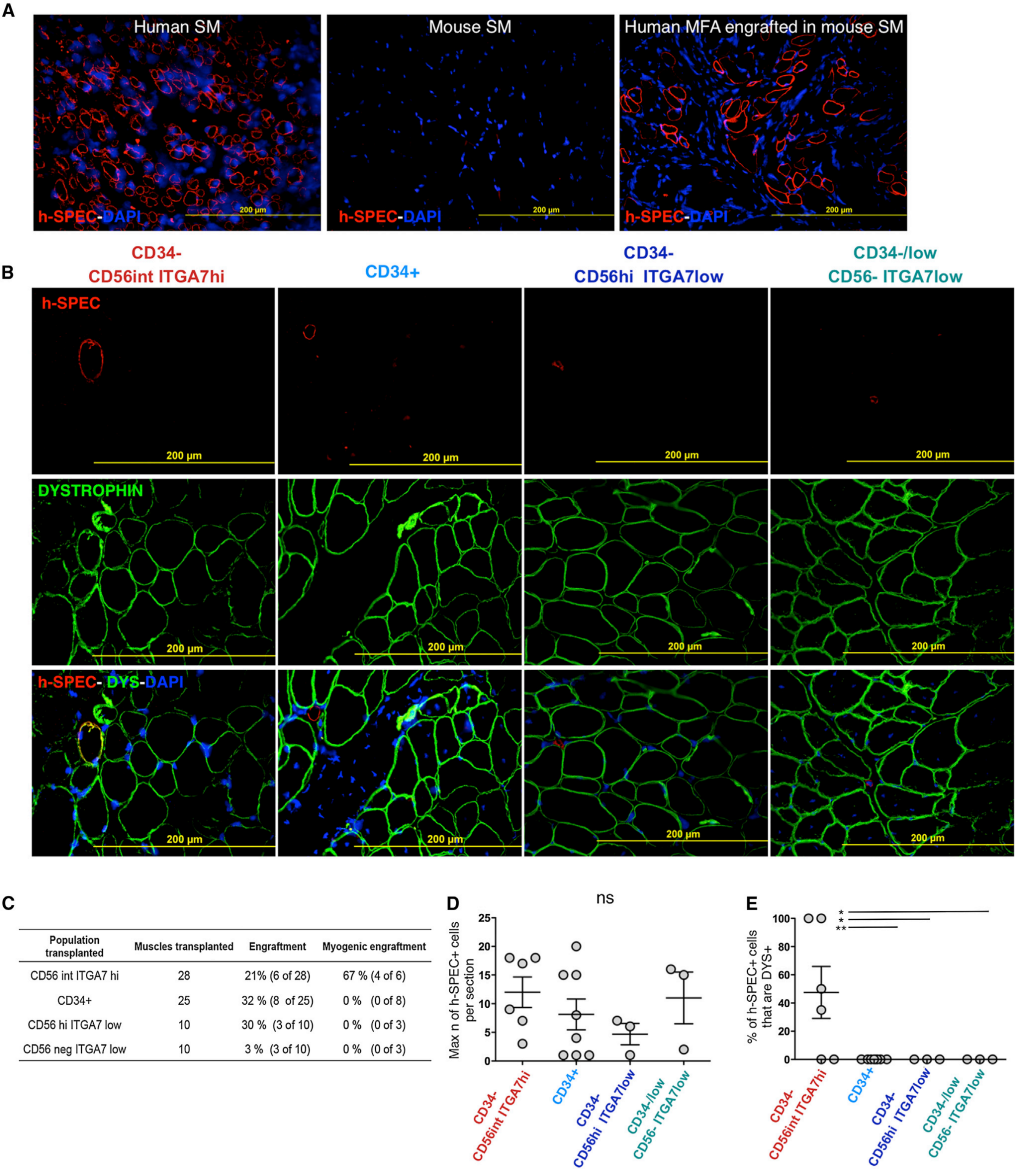
In Vivo Engraftment of Fetal hMFA Cell Subsets in Mouse Muscle Engrafted hMFA cells were detected in transplanted NSG mouse muscle by costaining for human species-specific h-SPECTRIN (h-SPEC, red), muscle-specific DYSTROPHIN (DYS, green), and DAPI (blue). (A) Species-specific staining for h-SPEC is strongly positive in human muscle (left) and absent in mouse muscle (middle). Unfractionated hMFA cells engrafted to form h-SPEC-positive cells in four out of four transplanted mice (right). (B) Engrafted fetal CD34^−^CD56^int^ITGA7^hi^ hMFA cells formed myofibers as demonstrated by costaining for h-SPEC and DYS. Engrafted CD34^+^, CD34^−^CD56^hi^ITGA7^low^, and CD34^−/low^CD56^−^ITGA7^low^ cells formed h-SPEC+, DYS− cells. (C) Engraftment efficiency was calculated for each subset as the number of muscles engrafted (i.e., h-SPEC+ cells present) of all muscles transplanted. (D) Engraftment was quantified as the maximum number of h-SPEC+ cells per engrafted muscle section. Data are mean ± SEM. (E) Myogenic engraftment was quantified as the percentage of h-SPEC+ cells that were also DYS+. Statistical significance was evaluated by unpaired, two-tailed t test (^∗^p < 0.05; ^∗∗^p < 0.001; ns, not significant). Data are mean ± SEM. See also Figure S5.

To assess the contributions of FACS-isolated fetal hMFA cells to muscle regeneration in vivo, we adapted previously published protocols to detect engraftment of unfractionated human myogenic cells in mouse muscle (Ehrhardt et al., 2007). Freshly isolated cells were injected directly into the cardiotoxin preinjured tibialis anterior muscles of immunodeficient nonobese diabetic severe combined immunodeficiency interleukin-2γ^−/−^ mice (NSG) mice, transplanted muscles were harvested 3–8 weeks after transplantation, and engraftment of human cells was detected by staining with antibodies against the human membrane protein SPECTRIN (h-SPECTRIN). H-SPECTRIN staining was strongly positive in fetal human muscle sections (Figure 5A, left panel) and uniformly absent in mouse muscle sections (Figure 5A, middle panel). Engraftment of unfractionated fetal hMFA cells, as evidenced by the presence of h-SPECTRIN^+^ cells on serial sections of transplanted muscles, was detected in four out of four transplanted mice (injected with 900,000 cells per mouse, isolated from two donors in two independent experiments; Figure 5A, right panel). To assess the in vivo myogenic activity of sorted fetal hMFA cell subsets, cells were isolated from 11 individual donors and transplanted into the cardiotoxin preinjured tibialis anterior muscles of NSG recipients. Engraftment by human cells, marked by staining with h-SPECTRIN, was detected in 6 of 28 muscles transplanted with CD34^−^CD56^int^ITGA7^hi^ fetal hMFA cells (37,000–100,000 cells injected), 8 of 25 muscles transplanted with CD34^+^ fetal cells (37,000–100,000 cells injected), three of ten muscles transplanted with CD34^−/low^CD56^−^ITGA7^low^ fetal cells (20,000–100,000 cells injected), and three of ten muscles transplanted with CD34^−^CD56^hi^ITGA7^low^ fetal cells (7,000–100,000 cells injected) (Figure 5B, top panels, and Figure 5C). Similar numbers of h-SPECTRIN-positive cells were detected in muscles engrafted with human CD34^−^CD56^int^ITGA7^hi^ cells (12 ± 3; mean ± SEM). CD34^+^ cells (8 ± 3; mean ± SEM), CD34^−/low^CD56^−^ITGA7^low^ cells (11 ± 5; mean ± SEM), or CD34^−^CD56^hi^ITGA7^low^ cells (5 ± 2; mean ± SEM) (Figure 5D).

We next evaluated myogenic engraftment of fetal hMFA cell subsets in transplanted mouse muscles by costaining for h-SPECTRIN (Figure 5B, top panels) and DYSTROPHIN (Figure 5B, middle panels), a membrane protein expressed in both mouse and human muscle fibers. Costaining of h-SPECTRIN and DYSTROPHIN (Figure 5B, bottom panels, and Figure S5) in four of six muscles engrafted with fetal human CD34^−^CD56^int^ITGA7^hi^ cells indicated that the transplanted cells underwent myogenic differentiation and contributed to the formation of mature fibers. A total of 35%–100% of h-SPECTRIN^+^ cells in these muscles coexpressed DYSTROPHIN. In contrast, none (0%) of the h-SPECTRIN+ cells detected in muscles engrafted with fetal CD34^+^ cells, CD34^−/low^CD56^−^ITGA7^low^ cells, or CD34^−^CD56^hi^ITGA7^low^ cells were DYSTROPHIN^+^ (Figure 5E). Thus, only fetal CD34^−^CD56^int^ITGA7^hi^ hMFA cells are capable of myogenic engraftment in mouse muscle.

### The Transcriptional Signatures of Fetal hMFA Cell Subsets Are Consistent with Lineage-Specific Differences in Their Differentiation Capacities

**Figure 6:**
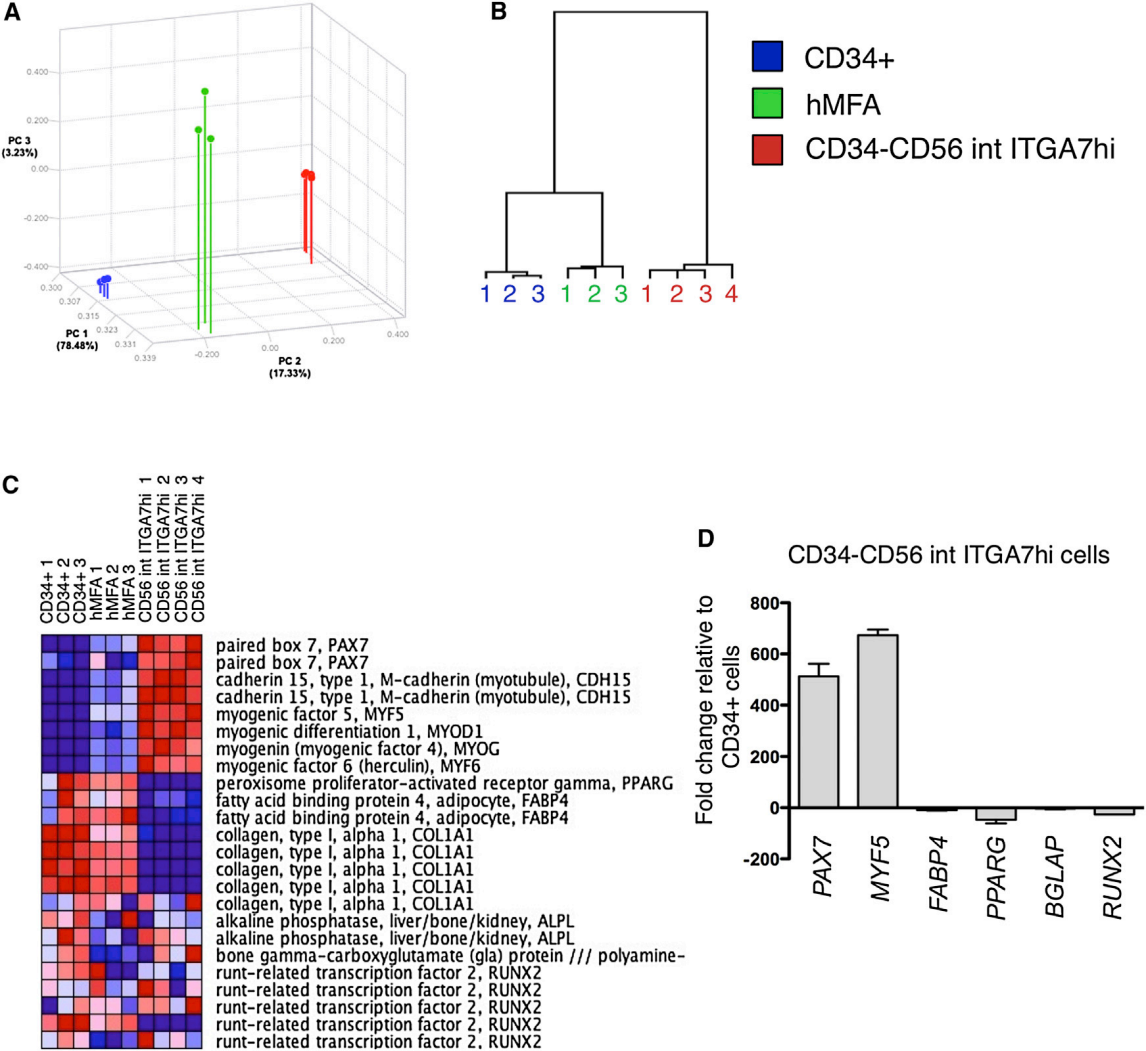
Distinct Transcriptional Signatures of Fetal CD34^−^CD56^int^ITGA7^hi^ and CD34^+^ hMFA Cells (A and B) PCA (A) and hierarchical clustering (B) demonstrate distinct gene expression signatures of CD34^+^ cells (blue, CD34^+^), CD34^−^CD56^int^ITGA7^hi^ cells (red, CD34^−^CD56^int^ITGA7^hi^), and unfractionated hMFA cells (green). Microarray analysis was performed using three to four (see B) biologically independent, freshly sorted CD34^−^CD56^int^ITGA7^hi^, CD34^+^, and unfractionated hMFA cell samples. (C) Microarray analyses demonstrated increased expression of muscle-lineage genes (*PAX7*, *MYF5*, *CDH15*, *MYOD*, *MYOG*) in CD34^−^CD56^int^ITGA7^hi^ cells and adipocyte-lineage genes (*PPARG* and *FABP4*) in CD34^+^ cells. Osteolineage genes (*COL1A1*, *ALPL*, *BGLAP*, and *RUNX2*) were present in fetal CD34^−^CD56^int^ITGA7^hi^ cells and CD34^+^ cells (blue, downregulated genes; red, upregulated genes). (D) Expression of *PAX7*, *MYF5*, *PPARG*, *FABP4*, *BGLAP*, and *RUNX2* (relative to *GAPDH*) was evaluated by qRT-PCR in fetal CD34^−^CD56^int^ITGA7^hi^ cells compared to CD34^+^ cells obtained from two biologically independent fetal CD34^−^CD56^int^ITGA7^hi^ and three biologically independent CD34^+^ cells samples. *PAX7* and *MYF5* levels are 512- to 670-fold greater, and *PPARG*, *FABP4*, *BGLAP*, and *RUNX2* levels are 8- to 60-fold lower, in CD34^−^CD56^int^ITGA7^hi^ hMFA cells as compared to CD34^+^ cells. Data are mean ± SD. See also Figure S6.

To gain deeper insights into the molecular underpinnings of CD34^−^CD56^int^ITGA7^hi^ cells and CD34^+^ adipogenic precursors within the fetal hMFA cell pool, the transcriptional profile of these functionally distinct populations, as compared to unfractionated fetal hMFA cells, was evaluated. Principal component analysis (PCA; Figure 6A) and hierarchical cluster analysis (Figure 6B) showed clustering of CD34^−^CD56^int^ITGA7^hi^ cells, CD34^+^ cells, and hMFA cells into three transcriptionally distinct populations. Comparison of CD34^−^CD56^int^ITGA7^hi^ cells (12.2% ± 4.3% of live fetal hMFA cells; Table S1) to unfractionated hMFA cells identified 5,686 differentially regulated probesets, and comparison of CD34^+^ cells (62.7% ± 7.8% of live fetal hMFA cells; Table S1) to unfractionated hMFA cells yielded 1,029 differentially regulated probesets (>1.5-fold difference up or down and p < 0.05). Notably, there was no overlap between these groups of differentially regulated genes. Ingenuity pathway analysis revealed that within the group of genes most highly upregulated in fetal CD34^−^CD56^int^ITGA7^hi^ cells as compared to CD34^+^ cells (>5-fold difference, p < 0.01, total 346 genes; Table S2), the 25 top-scoring functions involved muscle development, differentiation, or function (Table S3). Interestingly, within the group of genes most highly upregulated in CD34^+^ cells versus CD34^−^CD56^int^ITGA7^hi^ cells (>5-fold difference, p < 0.01, total 854 genes; Table S4), the seven top-scoring functions involved solid tumor malignancy (Table S5).

We also specifically analyzed expression by freshly isolated fetal CD34^−^CD56^int^ITGA7^hi^ cells and CD34^+^ cells of certain myogenic lineage (*PAX7*, *MYF5*, *M-CADHERIN/CDH15*, *MYOD*, *MYOG*), adipogenic lineage (*PPARG*, *FABP4*) and osteogenic lineage (*COL1A*, *ALPL*, *BGLAP*, *RUNX2*) genes in the microarray data set (Figure 6C). Expression of satellite cell markers (including the HGF receptor *MET*; Figure S6C) and myogenic genes was upregulated in fetal CD34^−^CD56^int^ITGA7^hi^ hMFA cells, whereas adipogenic genes were upregulated in fetal CD34^+^ cells (Figure 6C). Expression of osteolineage genes was detected in both CD34^−^CD56^int^ITGA7^hi^ cells and CD34^+^ cells (Figure 6C), consistent with the osteogenic activity of both populations. Finally, we confirmed differential expression of adipogenic, osteogenic, and myogenic lineage-specific genes (*PPARG*, *FABP4*, *BGLAP*, *RUNX2*, *PAX7*, and *MYF5*) in fetal CD34^−^CD56^int^ITGA7^hi^ and CD34^+^ hMFA cells by qRT-PCR (Figure 6D). Levels of *PPARG* (fold-change −47 ± 20), *FABP4* (fold-change −10 ± 2) and *BGLAP* (fold-change −4 ± 3) were reduced in CD34^−^CD56^int^ITGA7^hi^ cells compared to CD34^+^ cells. In contrast, *PAX7* (fold-change +513 ± 70) and *MYF5* (fold-change +674 ± 31) levels were increased in CD34^−^CD56^int^ITGA7^hi^ cells, consistent with their myogenic function. Thus, sorted CD34^−^CD56^int^ITGA7^hi^ cells and CD34^+^ cells from fetal human muscle possess transcriptional signatures highly consistent with their distinct differentiation potentials.

Finally, our microarray analyses identified a number of additional surface markers as differentially regulated in fetal CD34^−^CD56^int^ITGA7^hi^ and CD34^+^ hMFA cells, including increased levels of *MCAM* (Figure S6B), *CD144* (Figure S6B), and *PROMININ1* (CD133; Figure S6B) and decreased levels of *PDGFRA* (CD140a; previously reported to mark adipocyte precursors; Berry and Rodeheffer, 2013; Figure S6B) in CD34^−^CD56^int^ITGA7^hi^ as compared to CD34^+^ cells (Table S6).

### Adult Skeletal Muscle Shows Reduced Content of PAX7-Expressing CD34^−^CD56^int^ITGA7^hi^ hMFA Cells with Myogenic and Osteogenic Activity

**Figure 7:**
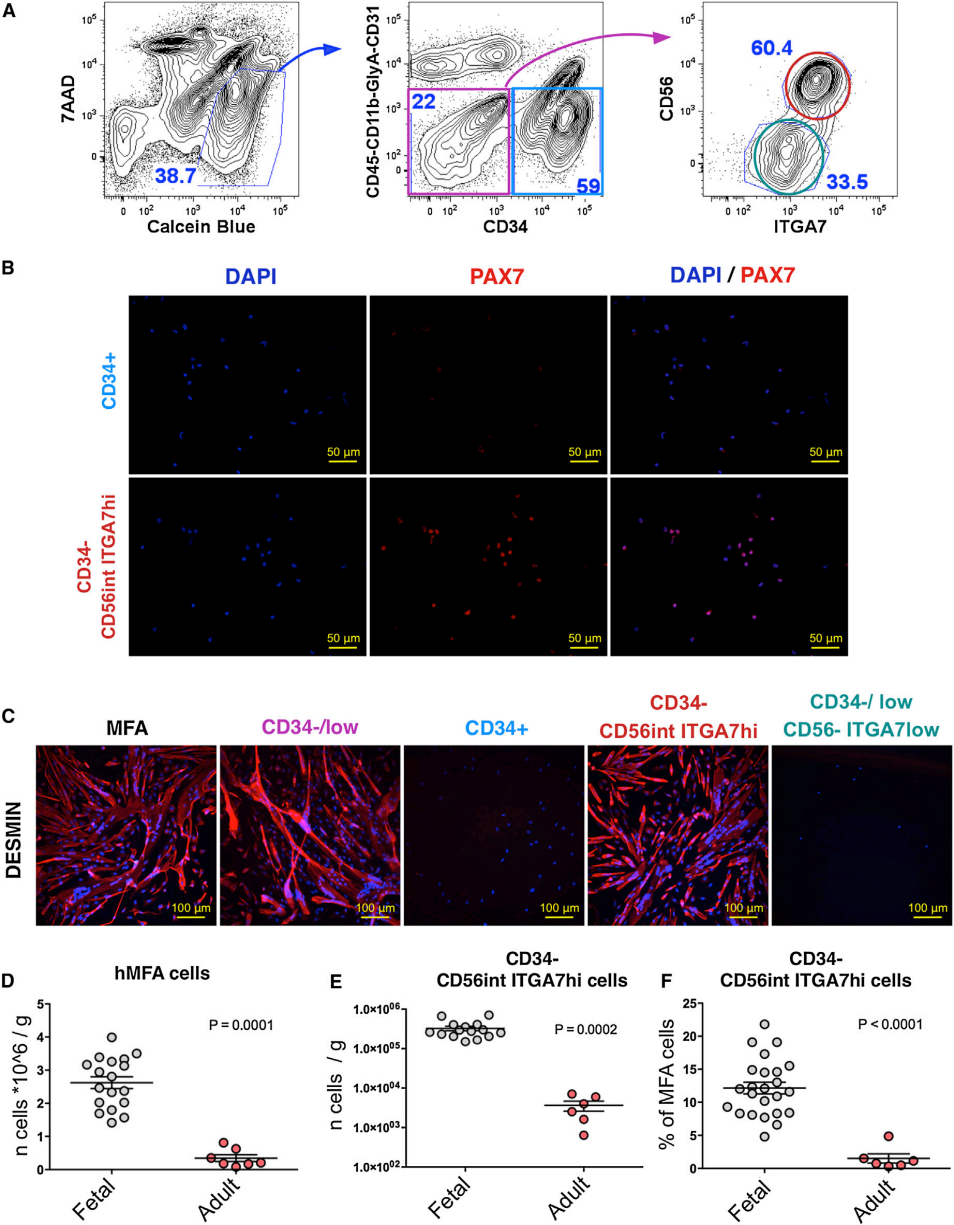
Adult CD34^−^CD56^int^ITGA7^hi^ hMFA Cells Are PAX7-Expressing Myogenic Progenitors (A) FACS gating strategy for isolation of CD45^−^CD11b^−^GlyA^−^CD31^−^CD34^−^CD56^int^ITGA7^hi^ cells within live (7AAD^−^ Calcein^+^) hMFA cells. (B) PAX7 expression (red) is enriched in CD34^−^CD56^int^ITGA7^hi^ hMFA cells (89% ± 7% [mean ± SD] Pax7^+^), as assessed by IF. Nuclei were marked by DAPI (blue). (C) Myogenic differentiation assays showed that in vitro myogenic activity is highly enriched in adult CD34^−^CD56^int^ITGA7^hi^ cells and absent from CD34^+^ and CD34^−/low^CD56^−^ITGA7^low^ cells. (D–F) Numbers of hMFA cells (D) or CD34^−^CD56^int^ITGA7^hi^ cells (E) per gram of tissue, and frequency of CD34^−^CD56^int^ITGA7^hi^ cells (F), were compared for adult and fetal muscle. Statistical significance was evaluated by unpaired, two-tailed t test. Data are mean ± SD. See also Figure S7.

To determine whether the surface marker combination we identified as marking PAX7-expressing osteogenic/myogenic progenitors in human fetal muscle would similarly mark progenitors in adult tissue, we evaluated differential expression of surface markers (Figure 7A), PAX7 enrichment (Figure 7B), myogenic differentiation (Figure 7C), and osteogenic differentiation (Figure S7) in hMFA cells obtained from discarded human adult muscle.

FACS analysis indicated clear separation of CD34^+^ and CD34^−/low^ subsets within the pool of viable CD45^−^CD11b^−^GlyA^−^CD31^−^ adult hMFA cells (Figure 7A). As in fetal muscle, all myogenic activity was contained within the CD34^−/low^ subset of adult hMFA cells, whereas CD34^+^ cells were uniformly nonmyogenic (Figure 7C). However, within the CD34^−/low^ adult hMFA cell pool, expression of CD56 and ITGA7 discriminated only two cell populations: CD34^−/low^CD56^−^ITGA7^low^ and CD34^−^CD56^int^ITGA7^hi^ cells. The CD34^−^CD56^hi^ITGA7^low^ subset detected in fetal muscle was not present in adult muscle (Figure 7A). We confirmed selective enrichment of PAX7-expressing cells (89% ± 7%, mean ± SD; Figure 7B) and in vitro myogenic activity (Figure 7C, second panel from right) in adult CD34^−^CD56^int^ITGA7^hi^ hMFA cells. Finally, analogous to fetal cells, adult CD34^−^CD56^int^ITGA7^hi^ cells exhibited osteogenic activity (Figure S7A) and lacked adipogenic potential (Figure S7B) in vitro, in addition to their myogenic function. Thus, CD34^−^CD56^int^ITGA7^hi^ hMFA cells isolated from adult muscle, similar to cells of the same marker phenotype obtained from fetal muscle, are PAX7-expressing cells with osteogenic/myogenic activity. However, total hMFA cell numbers were significantly lower in adult muscle (mean of 0.4 × 10^6^ [adult] versus 2.5 × 10^6^ [fetal] hMFA cells per gram muscle; p = 0.0001; Figure 7D), and the percentage of CD34^−^CD56^int^ITGA7^hi^ cells among hMFA cells was also lower (mean of 12.2% ± 1.7% [fetal] versus 1.5% ± 1.7% [adult]; p < 0.0001; Figure 7F). This translated into an ∼2-log reduction in the total number of CD34^−^CD56^int^ITGA7^hi^ hMFA cells in adult as compared to fetal muscle (mean 3.3 × 10^5^ [fetal] versus 3.6 × 10^3^ [adult] cells per gram of muscle; p = 0.0002; Figure 7E). Decreasing muscle progenitor frequency with age in human muscle is consistent with previously published findings in the mouse (Conboy et al., 2003).

## Discussion

Recent advances enabling the prospective isolation of mouse satellite cells have facilitated mechanistic analyses of their myogenic function. For example, the ability to clonally sort satellite cells with high purity made possible the demonstration that these cells can undergo asymmetric division (Kuang et al., 2007, Rocheteau et al., 2012) and repopulate the satellite cell niche in vivo (Cerletti et al., 2008). While findings in mouse models are often extrapolated to human biology, whether mouse and human myogenic cells exhibit fully equivalent properties may still be questioned, particularly given significant phenotypic discrepancies in several mouse models of human muscle disease (Bulfield et al., 1984). All of these issues can be addressed through the establishment of robust methods for direct purification of human muscle progenitors.

Previous work by Pisani et al. demonstrated the utility of the sialomucin CD34 to enrich for myogenic cells within the CD34^−^ subset of magnetically separated cells in adult muscle (Pisani et al., 2010b), consistent with immunohistochemical studies reporting the absence of CD34 in adult human muscle cells located in the satellite cell position (Lecourt et al., 2010). Pisani et al. also noted mixed myogenic and adipogenic activity within CD34^+^ adult muscle cells, which showed differential expression of CD56 (Pisani et al., 2010a). Findings from our study confirm that CD34 distinguishes myogenic and nonmyogenic cells within the nonhematopoietic, nonendothelial (CD45^−^CD11b^−^GlyA^−^CD31^−^) hMFA cell pool in both fetal and adult tissue: CD34^+^ cells are PAX7-negative, adipogenic cells that do not possess any myogenic activity, whereas within the CD45^−^CD11b^−^GlyA^−^CD31^−^CD34^−/low^ subset, selection of CD56^int^ITGA7^hi^ cells yields a highly enriched population of PAX7-expressing, robustly myogenic progenitors. Yet, it is important to note that these FACS-based strategies pertain to cells isolated from fresh muscle only. Sorted cells may undergo marked changes in their surface marker profiles during ex vivo culture, and it is unclear if our protocols are applicable to cells that have undergone expansion/differentiation in culture.

Fluorescence-activated cell-sorted fetal human CD34^−^CD56^int^ITGA7^hi^ cells engraft in mouse muscle to form new myofibers, albeit at low efficiency (Figures 5B–5F). Low-level engraftment of human cells into mouse tissue is not unexpected, as similar outcomes have been observed for other human, tissue-specific stem and progenitor cells upon transfer into immune-compromised mice (Doulatov et al., 2012, Racki et al., 2010). Unfortunately, given the relatively sparse presence of human myofibers in this system, we were unable to establish conditions to reliably detect costaining for PAX7 and human species-specific nuclear antigens on engrafted mouse muscle sections. We therefore were unable to determine if fluorescence-activated cell sorted fetal human CD34^−^CD56^int^ITGA7^hi^ cells can repopulate the PAX7-expressing satellite cell pool in vivo. We also were unable to test the in vivo engraftment potential of fluorescence-activated cell-sorted progenitors from adult human muscle, given the low yield of cells that could be obtained from adults (Figure 7E).

Interestingly, enrichment of PAX7 expression and myogenic activity within the CD34^−^ compartment in human muscle stands in contrast to immunophenotyping studies in mouse muscle, which localize myogenic activity to the CD34^+^ subset of mouse MFA cells (Beauchamp et al., 2000, Conboy et al., 2010, Montarras et al., 2005, Sacco et al., 2008, Sherwood et al., 2004). Species-specific differences in CD34 expression have also been noted in other somatic stem cell populations, including hematopoietic stem cells (HSCs), which are CD34^+^ in adult human bone marrow and CD34^−^ in adult mouse bone marrow (Okuno et al., 2002, Osawa et al., 1996). Such differences appear to arise from the presence of species-specific upstream regulatory elements, which differentially regulate CD34 gene transcription in mouse and human cells (Okuno et al., 2002).

Cell surface markers that have proved useful for the isolation of mouse PAX7^+^ satellite cells include ITGA7 (Pasut et al., 2012, Sacco et al., 2008), CXCR4 and β1 INTEGRIN (Sherwood et al., 2004), and VCAM1 (Chakkalakal et al., 2012). Our studies indicate that PAX7-expressing cells in fetal and adult human muscle coexpress ITGA7 and CD56, although neither of these markers alone is sufficient to distinguish these cells. This is consistent with ITGA7 and CD56 expression by human myogenic progenitors derived from PAX7-expressing induced pluripotent stem cells (Darabi et al., 2012). Clear expression of CXCR4 was observed in adult and fetal PAX7-expressing cells (Figure S6A), and β1-INTEGRIN was detected on 90% of hMFA cells (Figure 1B). Additional surface marker analyses, focusing particularly on those previously linked to mouse and/or human myogenic precursors (Cerletti et al., 2012, Darabi et al., 2012, Lecourt et al., 2010, Zheng et al., 2007), revealed increased expression of *MCAM* (CD146) and *CD44* in human fetal CD34^−^CD56^int^ITGA7^hi^ cells as compared to CD34^+^ cells (Figure S6B). Finally, our microarray analyses suggest that *PROMININ1* (CD133) and *PDGFRA* (CD140a; previously reported to mark adipocyte precursors; Berry and Rodeheffer, 2013) could also be useful in distinguishing human myogenic progenitors, as they are differentially expressed in fetal CD34^−^CD56^int^ITGA7^hi^ cells and CD34^+^ cells (Figure S6C; Table S6).

MFA cells obtained from mouse and human muscle were previously shown to exhibit osteogenic activity (Glass et al., 2011, Hashimoto et al., 2008, Lecourt et al., 2010, Oishi et al., 2013). In our studies, both fetal and adult PAX7-expressing CD34^−^CD56^int^ITGA7^hi^ cells formed AR-positive calcium deposits and expressed osteogenic lineage genes under osteogenic conditions, consistent with prior reports of osteogenic differentiation potential within the pool of muscle cells containing PAX7^+^ progenitors (Hashimoto et al., 2008, Ozeki et al., 2006). Clonal assays revealed that the osteogenic activity of human fetal CD34^−^CD56^int^ITGA7^hi^ hMFA cells is unlikely to be attributable to contamination by other cells, as the majority of clone-sorted cells exhibited bipotent myogenic and osteogenic activity. Future studies are needed to investigate adult muscle progenitor bipotency and delineate the events that trigger possible osteogenic differentiation of human CD34^−^CD56^int^ITGA7^hi^ cells, their possible contributions to normal bone regeneration, and their relationship to other mesenchymal precursor cells.

In contrast to fetal CD34^−^CD56^int^ITGA7^hi^ cells, which exhibit robust myogenic activity and lack adipogenic potential, human fetal CD34^+^ cells are adipogenic and lack myogenic capacity. While our studies evaluated the ability of these cells to form white adipocytes, a previous report indicates that CD34^+^ cells in fetal and adult human muscle contain brown adipogenic activity as well (Crisan et al., 2008). Gene expression profiling confirms profound differences in the transcriptional signatures of fetal human CD34^−^CD56^int^ITGA7^hi^ and CD34^+^ hMFA cells, with increased expression of muscle lineage genes in CD34^−^CD56^int^ITGA7^hi^ cells and increased expression of adipogenic genes in CD34^+^ cells. Given their differentiation profile, we speculate that CD34^+^ hMFA cells may represent the human counterparts of the fibroadipogenic precursor (FAP) population in mouse muscle (Joe et al., 2010, Uezumi et al., 2010), an important subset of nonmyogenic cells that appears to enhance muscle regenerative capacity (Joe et al., 2010).

In summary, we report functionally distinct cell populations within the hMFA cell pool in fetal and adult muscle and provide a specific method for the prospective isolation of purified PAX7^+^ cells from human muscle. We anticipate that this technology will facilitate novel insights into human muscle homeostasis, aging, and disease. Phenotypically distinct cells with myogenic progenitor function also represent promising targets for muscle regenerative cell therapy and could conceivably be used to treat a variety of diseases, including muscular dystrophy and muscle injuries.

## Experimental Procedures

### Human Skeletal Muscle Specimens

Human fetal muscle was obtained from 20- to 23-week-gestation fetuses and adult muscle from deceased volunteers or discarded during surgery (Table S1). Use of human tissues was approved by relevant institutional review boards.

### Isolation of hMFA Cells

hMFA cells were isolated by two-step enzymatic digestion and mechanical dissociation as per previously published protocols (Sherwood et al., 2004).

### Antibody Staining and FACS

Primary and secondary antibodies used for FACS are listed in Table S7. All cell populations were sorted twice to maximize purity.

### PAX7 IF and Quantification

hMFA cell subsets were sorted directly into 40 μl of PBS spotted on a glass slide (5 × 10^3^ cells per slide) according to protocols adapted from (Ema et al., 2006). Sorted cells were stained with PAX7 (DSHB, 10 μg/ml).

### Myogenic Differentiation Assay

hMFA cell subsets were sorted in 96-well plates, expanded in myogenic growth medium for 7 days, transitioned into differentiation medium for 4–5 days, and fixed and stained with DESMIN antibody (clone D33, M0760, titer 1:50; Dako).

### Adipogenic Differentiation Assay

hMFA cell subsets were sorted in 96-well plates, expanded in adipogenic growth medium until confluent (13–14 days), transitioned into adipogenic induction medium for 3 days, placed in differentiation medium for 4 days, and fixed and stained with ORO (Sigma).

### Osteogenic Differentiation Assay

hMFA cell subsets were sorted in 96-well plates, expanded in preadipocyte medium (PM-1, ZenBio) + 25 ng/ml basic fibroblast growth factor (Sigma) until confluent (13–14 days), transitioned into osteoblast differentiation medium (OB-1, ZenBio) for 14 days, and fixed and stained with 2% AR (Sigma).

### Clonal Cell Culture

Fetal hMFA cell subpopulations were sorted at 1 cell per well in 96-well plates in myogenic growth medium. After 9–10 days, the number of wells with visible cell growth was determined. Cells were kept in myogenic growth conditions until confluent and passaged into myogenic or osteogenic differentiation assays.

### Transplantation Studies

NSG mice (Jackson Laboratory) were bred and maintained at Joslin Diabetes Center under Institutional Animal Care and Use Committee-approved protocols. The tibialis anterior (TA) muscle of 6- to 8-week-old NSG mice was conditioned 24 hr prior to transplantation by injection of 25 μl (0.03 mg/ml) of Naja mossambica mossambica cardiotoxin (CTX, Sigma). Recipient muscles were harvested 3–8 weeks after transplantation. Engraftment was evaluated by IF staining of 7 μm sections for h-SPECTRIN (human species-specific) and DYSTROPHIN. Tissue was blocked using Papain-digested RAM antibodies supplemented with goat Fc antibodies at 5 μg/ml and 5% fetal bovine serum according to previously published protocols (Ehrhardt et al., 2007).

### Microarray Analysis

Total RNA was extracted using TRIzol labeled and hybridized to Affymetrix microarrays (Human Genome U133 Plus 2.0). Raw data were normalized and differentially regulated probesets were identified using GenePattern. Hierarchical clustering was performed in GenePattern (Broad Institute), PCA using 3D-PCA, and pathway analysis using Ingenuity.

### PCR

Total RNA was extracted using TRIzol, reverse transcribed using Superscript III First-Strand Synthesis System (Invitrogen). qRT-PCR was performed using an AV7900 PCR system (Applied Biosystems) and TaqMan Gene Expression Assays (Invitrogen).

### Statistics

Statistical analysis was performed using two-tailed Student’s t test for unpaired data when appropriate. p values are indicated with asterisks (^∗^p < 0.05, ^∗∗^p < 0.001, and ^∗∗∗^p < 0.0001) and NS (not significant).

For additional details regarding experimental procedures, please see the Supplemental Experimental Procedures.

## Accession Numbers

The microarray data reported in this paper were deposited in the NCBI Gene Expression Omnibus under accession number GSE44227.

## Supplemental Information

Document S1. Supplemental Experimental Procedures and Figures S1–S7

Table S1. List of Human Fetal and Adult Skeletal Muscle Samples Used in This Study, Related to Figures 1, 2, 3, 4, 5, and 6

Table S2. Genes Upregulated in Fetal CD34^−^CD56^int^ITGA7^hi^ versus CD34^+^ Cells, Related to Figure 6 Greater than 5-fold, p < 0.01.

Table S3. Top Functions among Genes Upregulated in Fetal CD34^−^CD56^int^ITGA7^hi^ versus CD34^+^ Cells as Determined by Ingenuity Pathway Analysis

Table S4. Genes Upregulated in Fetal CD34^+^ versus CD34^−^CD56^int^ITGA7^hi^ Cells, Related to Figure 6 Greater than 5-fold, p < 0.01.

Table S5. Top Functions among Genes Upregulated in Fetal CD34^+^ versus CD34^−^CD56^int^ITGA7^hi^ Cells as Determined by Ingenuity Pathway Analysis, Related to Figure 6

Table S6. Cell Membrane Molecules Differentially Regulated in CD34^−^CD56^int^ITGA7^hi^ versus CD34^+^ Cells, Related to Figure 6

Table S7. List of Antibodies Used for Flow Cytometry and FACS of hMFA Cell Subsets, Related to Figures 1, 2, 3, 4, 5, 6, and 7

## References

[bib1] Andukuri A., Sohn Y.D., Anakwenze C.P., Lim D.J., Brott B.C., Yoon Y.S., Jun H.W. (2013). Enhanced human endothelial progenitor cell adhesion and differentiation by a bioinspired multifunctional nanomatrix. Tissue Eng. Part C Methods.

[bib2] Beauchamp J.R., Heslop L., Yu D.S., Tajbakhsh S., Kelly R.G., Wernig A., Buckingham M.E., Partridge T.A., Zammit P.S. (2000). Expression of CD34 and Myf5 defines the majority of quiescent adult skeletal muscle satellite cells. J. Cell Biol..

[bib3] Berry R., Rodeheffer M.S. (2013). Characterization of the adipocyte cellular lineage in vivo. Nat. Cell Biol..

[bib4] Bosnakovski D., Xu Z., Li W., Thet S., Cleaver O., Perlingeiro R.C., Kyba M. (2008). Prospective isolation of skeletal muscle stem cells with a Pax7 reporter. Stem Cells.

[bib5] Bulfield G., Siller W.G., Wight P.A., Moore K.J. (1984). X chromosome-linked muscular dystrophy (mdx) in the mouse. Proc. Natl. Acad. Sci. USA.

[bib6] Cerletti M., Jurga S., Witczak C.A., Hirshman M.F., Shadrach J.L., Goodyear L.J., Wagers A.J. (2008). Highly efficient, functional engraftment of skeletal muscle stem cells in dystrophic muscles. Cell.

[bib7] Cerletti M., Jang Y.C., Finley L.W., Haigis M.C., Wagers A.J. (2012). Short-term calorie restriction enhances skeletal muscle stem cell function. Cell Stem Cell.

[bib8] Chakkalakal J.V., Jones K.M., Basson M.A., Brack A.S. (2012). The aged niche disrupts muscle stem cell quiescence. Nature.

[bib9] Conboy I.M., Conboy M.J., Smythe G.M., Rando T.A. (2003). Notch-mediated restoration of regenerative potential to aged muscle. Science.

[bib10] Conboy M.J., Cerletti M., Wagers A.J., Conboy I.M. (2010). Immuno-analysis and FACS sorting of adult muscle fiber-associated stem/precursor cells. Methods Mol. Biol..

[bib11] Crisan M., Casteilla L., Lehr L., Carmona M., Paoloni-Giacobino A., Yap S., Sun B., Léger B., Logar A., Pénicaud L. (2008). A reservoir of brown adipocyte progenitors in human skeletal muscle. Stem Cells.

[bib12] Darabi R., Arpke R.W., Irion S., Dimos J.T., Grskovic M., Kyba M., Perlingeiro R.C. (2012). Human ES- and iPS-derived myogenic progenitors restore DYSTROPHIN and improve contractility upon transplantation in dystrophic mice. Cell Stem Cell.

[bib13] Doulatov S., Notta F., Laurenti E., Dick J.E. (2012). Hematopoiesis: a human perspective. Cell Stem Cell.

[bib14] Ehrhardt J., Brimah K., Adkin C., Partridge T., Morgan J. (2007). Human muscle precursor cells give rise to functional satellite cells in vivo. Neuromuscul. Disord..

[bib15] Ema H., Morita Y., Yamazaki S., Matsubara A., Seita J., Tadokoro Y., Kondo H., Takano H., Nakauchi H. (2006). Adult mouse hematopoietic stem cells: purification and single-cell assays. Nat. Protoc..

[bib16] Fukada S., Higuchi S., Segawa M., Koda K., Yamamoto Y., Tsujikawa K., Kohama Y., Uezumi A., Imamura M., Miyagoe-Suzuki Y. (2004). Purification and cell-surface marker characterization of quiescent satellite cells from murine skeletal muscle by a novel monoclonal antibody. Exp. Cell Res..

[bib17] Glass G.E., Chan J.K., Freidin A., Feldmann M., Horwood N.J., Nanchahal J. (2011). TNF-alpha promotes fracture repair by augmenting the recruitment and differentiation of muscle-derived stromal cells. Proc. Natl. Acad. Sci. USA.

[bib18] Hashimoto N., Kiyono T., Wada M.R., Umeda R., Goto Y., Nonaka I., Shimizu S., Yasumoto S., Inagawa-Ogashiwa M. (2008). Osteogenic properties of human myogenic progenitor cells. Mech. Dev..

[bib19] Joe A.W., Yi L., Natarajan A., Le Grand F., So L., Wang J., Rudnicki M.A., Rossi F.M. (2010). Muscle injury activates resident fibro/adipogenic progenitors that facilitate myogenesis. Nat. Cell Biol..

[bib20] Kuang S., Kuroda K., Le Grand F., Rudnicki M.A. (2007). Asymmetric self-renewal and commitment of satellite stem cells in muscle. Cell.

[bib21] Lecourt S., Marolleau J.P., Fromigué O., Vauchez K., Andriamanalijaona R., Ternaux B., Lacassagne M.N., Robert I., Boumédiene K., Chéreau F. (2010). Characterization of distinct mesenchymal-like cell populations from human skeletal muscle in situ and in vitro. Exp. Cell Res..

[bib22] McKinnell I.W., Ishibashi J., Le Grand F., Punch V.G., Addicks G.C., Greenblatt J.F., Dilworth F.J., Rudnicki M.A. (2008). Pax7 activates myogenic genes by recruitment of a histone methyltransferase complex. Nat. Cell Biol..

[bib23] Montarras D., Morgan J., Collins C., Relaix F., Zaffran S., Cumano A., Partridge T., Buckingham M. (2005). Direct isolation of satellite cells for skeletal muscle regeneration. Science.

[bib24] Oishi T., Uezumi A., Kanaji A., Yamamoto N., Yamaguchi A., Yamada H., Tsuchida K. (2013). Osteogenic differentiation capacity of human skeletal muscle-derived progenitor cells. PLoS ONE.

[bib25] Okuno Y., Iwasaki H., Huettner C.S., Radomska H.S., Gonzalez D.A., Tenen D.G., Akashi K. (2002). Differential regulation of the human and murine CD34 genes in hematopoietic stem cells. Proc. Natl. Acad. Sci. USA.

[bib26] Osawa M., Hanada K., Hamada H., Nakauchi H. (1996). Long-term lymphohematopoietic reconstitution by a single CD34-low/negative hematopoietic stem cell. Science.

[bib27] Ozeki N., Lim M., Yao C.C., Tolar M., Kramer R.H. (2006). alpha7 integrin expressing human fetal myogenic progenitors have stem cell-like properties and are capable of osteogenic differentiation. Exp. Cell Res..

[bib28] Pasut A., Oleynik P., Rudnicki M.A. (2012). Isolation of muscle stem cells by fluorescence activated cell sorting cytometry. Methods Mol. Biol..

[bib29] Pisani D.F., Clement N., Loubat A., Plaisant M., Sacconi S., Kurzenne J.Y., Desnuelle C., Dani C., Dechesne C.A. (2010). Hierarchization of myogenic and adipogenic progenitors within human skeletal muscle. Stem Cells.

[bib30] Pisani D.F., Dechesne C.A., Sacconi S., Delplace S., Belmonte N., Cochet O., Clement N., Wdziekonski B., Villageois A.P., Butori C. (2010). Isolation of a highly myogenic CD34-negative subset of human skeletal muscle cells free of adipogenic potential. Stem Cells.

[bib31] Racki W.J., Covassin L., Brehm M., Pino S., Ignotz R., Dunn R., Laning J., Graves S.K., Rossini A.A., Shultz L.D., Greiner D.L. (2010). NOD-scid IL2rgamma(null) mouse model of human skin transplantation and allograft rejection. Transplantation.

[bib32] Rocheteau P., Gayraud-Morel B., Siegl-Cachedenier I., Blasco M.A., Tajbakhsh S. (2012). A subpopulation of adult skeletal muscle stem cells retains all template DNA strands after cell division. Cell.

[bib33] Sacco A., Doyonnas R., Kraft P., Vitorovic S., Blau H.M. (2008). Self-renewal and expansion of single transplanted muscle stem cells. Nature.

[bib34] Seale P., Sabourin L.A., Girgis-Gabardo A., Mansouri A., Gruss P., Rudnicki M.A. (2000). Pax7 is required for the specification of myogenic satellite cells. Cell.

[bib35] Sherwood R.I., Christensen J.L., Conboy I.M., Conboy M.J., Rando T.A., Weissman I.L., Wagers A.J. (2004). Isolation of adult mouse myogenic progenitors: functional heterogeneity of cells within and engrafting skeletal muscle. Cell.

[bib36] Tanaka O., Shinohara H., Oguni M., Yoshioka T. (1995). Ultrastructure of developing muscle in the upper limbs of the human embryo and fetus. Anat. Rec..

[bib37] Tanaka K.K., Hall J.K., Troy A.A., Cornelison D.D., Majka S.M., Olwin B.B. (2009). Syndecan-4-expressing muscle progenitor cells in the SP engraft as satellite cells during muscle regeneration. Cell Stem Cell.

[bib38] Uezumi A., Fukada S., Yamamoto N., Takeda S., Tsuchida K. (2010). Mesenchymal progenitors distinct from satellite cells contribute to ectopic fat cell formation in skeletal muscle. Nat. Cell Biol..

[bib39] Wang Y.X., Rudnicki M.A. (2012). Satellite cells, the engines of muscle repair. Nat. Rev. Mol. Cell Biol..

[bib40] Zheng B., Cao B., Crisan M., Sun B., Li G., Logar A., Yap S., Pollett J.B., Drowley L., Cassino T. (2007). Prospective identification of myogenic endothelial cells in human skeletal muscle. Nat. Biotechnol..

