# Supplementary material for: Isolation of Progenitors that Exhibit Myogenic/Osteogenic Bipotency In Vitro by Fluorescence-Activated Cell Sorting from Human Fetal Muscle
Source: Stem Cell Reports. 2014 Jan 14;2(1):92–106. doi: 10.1016/j.stemcr.2013.12.006 (PMC3966115; doi:10.1016/j.stemcr.2013.12.006)
Supplement: Document S1. Supplemental Experimental Procedures and Figures S1–S7 [file mmc1.pdf]

Stem Cell Reports, Volume *2*

Supplemental Information

Isolation of Progenitors that Exhibit Myogenic/ Osteogenic Bipotency In Vitro by Fluorescence-Activated Cell Sorting from Human Fetal Muscle

Alessandra Castiglioni, Simone Hettmer, Matthew D. Lynes, Tata Nageswara Rao, Daria Tchessaolova, Indranil Sinha, Bernard T. Lee, Yu-Hua Tseng, and Amy J. Wagers

Inventory of Supplemental Information

Figure S1. Expression of cell surface antigens by flow cytometry in live ($Pi^{-}Ca^{+}$ or $7AAD^{-}Ca^{+}$) human fetal MFA cells (linked to Figure 1).

Figure S2. PAX7 expression by immunofluorescence in prospectively isolated fetal hMFA cell subsets (linked to Figure 2).

Figure S3. Purity of FACS-sorted fetal hMFA cell subsets (linked to Figure 4).

Figure S4. Myogenic differentiation capacity of hMFA cell subsets under adipogenic (a) or osteogenic (b) conditions in vitro (linked to Figure 3).

Figure S5. In vivo myogenic engraftment of fetal human $CD34^{-}CD56^{int}ITGA7^{hi}$ hMFA cells (linked to Figure 5).

Figure S6. Additional cell surface markers for human myogenic/osteogenic progenitor cells in muscle (linked to Figure 6).

Figure S7. Osteogenic and adipogenic differentiation capacity of adult hMFA subsets (linked to Figure 7).

Table S1. List of human fetal and adult skeletal muscle samples used in this study (linked to Figures 1-6).

Table S2. Genes upregulated in fetal $CD34^{-}CD56^{int}ITGA7^{hi}$ versus $CD34^{+}$ cells (> 5-fold, p< 0.01; linked to Figure 6).

Table S3. Top functions among genes upregulated in fetal $CD34^{-}CD56^{int}ITGA7^{hi}$ versus $CD34^{+}$ cells as per Ingenuity pathway analysis (linked to Figure 6).

Table S4. Genes upregulated in fetal $CD34^{+}$ versus $CD34^{-}CD56^{int}ITGA7^{hi}$ cells (> 5-fold, p< 0.01; linked to Figure 6).

Table S5. Top functions among genes upregulated in fetal $CD34^{+}$ versus $CD34^{-}CD56^{int}ITGA7^{hi}$ cells as per Ingenuity pathway analysis (linked to Figure 6).

Table S7. List of antibodies used for flow cytometry analysis and prospective FACS sorting of discrete hMFA subsets (linked to Figure 1-7).

SUPPLEMENTAL EXPERIMENTAL PROCEDURES, FIGURES AND TABLES.

Supplemental Experimental Procedures

Human Skeletal Muscle Specimens

Human fetal muscle specimens (Table S1) were obtained from aborted 20-23 week gestation fetuses through Advanced Bioscience Resources (ABR, Alameda, CA). The presence of mature myotubes in >11-12 weeks gestation human fetal muscle was previously reported (Ehrhardt et al., 2007; Tanaka et al., 1995). Human adult muscle specimens (Table S1) were obtained from deceased volunteers through the National Development and Research Institutes (NDRI, Philadelphia, PA) or from skeletal muscle tissue that was discarded during routine surgery. Informed consent was obtained prior to tissue procurement. Use of human tissues was approved by the Institutional Review Boards at the Joslin Diabetes Center and the Beth Israel Deaconess Medical Center (CHS#08-20, CHS#08-21, BI 2012-P-000281/1).

Isolation of human Myofiber-Associated (hMFA) Cells

Adult and fetal human skeletal muscle was dissected at the donor center and maintained in DMEM at 4°C for up to 24 hours during transportation. Further processing of tissues took place at the Joslin Diabetes Center in Boston, MA. To isolate MFA cells, muscle specimens were digested in DMEM containing 0.2% Collagenase type II (Invitrogen, Carlsbad, CA) for 60 minutes at 37°C in a shaking waterbath, mechanically dissociated using flame-polished glass pipettes and incubated at 37°C for 12 minutes x 3 to allow fibers to settle by gravity. MFA cells were subsequently liberated from fibers by 30-minute digestion in F10 containing 0.0125% Collagenase type II (Invitrogen) and 0.05% Dispase (Invitrogen). Red blood cells were lysed by exposure to 0.15M Ammonium Chloride and 0.01M Potassium Chloride for 3 minutes at 4°C. Cells were counted using Trypan Blue to exclude dead cells, frozen in 90%

fetal bovine serum (FBS) supplemented with 10% DMSO or Cryostor (Sigma St. Louis, MO) and stored in liquid nitrogen for future use.

Antibody staining and fluorescence activated cell sorting (FACS).

Primary and secondary antibodies used for FACS are listed in Table S7. Antibody titers were optimized using primary, unfractionated human muscle cells. Cells were incubated in HBSS (Invitrogen) containing 10% rat IgG for 20 minutes at 4°C prior to antibody staining for FACS. Antibody staining was performed for 20 min on ice in HBSS (Invitrogen) + 2% FBS (staining medium, SM). Calcein Blue (Invitrogen), 7AAD (Becton Dickinson) and PI were used to distinguish live cells. FACS was performed using a FACSARIA (Becton Dickinson, Franklin Lakes, NJ). All cell populations were sorted twice to maximize purity. The purity of sorted cells was determined in aliquots of sorted cells for 4 biologically independent samples per MFA cell subset (Fig S3e).

PAX7 immunofluorescence and quantification

HMFA cells were sorted directly into 40μl of PBS spotted on a glass slide ($5x10^3$ cells per slide). Cells were dried briefly at room temperature (RT), fixed in 4% paraformaldehyde (PFA) for 20 minutes at RT and blocked prior to staining in 100% normal goat serum at RT for 1 hour. Cells were stained with primary antibody (monoclonal mouse anti-PAX7 antibody, DSHB, 10 μg/ml) at 4°C overnight and with secondary antibody (goat anti-mouse Alex Fluor 594 conjugate, Invitrogen, 1:200) for one hour at RT. Slides were coverslipped using Vectashield mounting media with DAPI to stain nuclei. Immunofluorescent labeling was analyzed by fluorescence microscopy using an Olympus BX60 upright microscope at 20-40X.

Myogenic Differentiation Assay

Human MFA cell subpopulations were sorted at 1 x 10^3 cells/ well in 96 well plates, coated with 2% Matrigel (BD). Cells were expanded for 7 days in myogenic growth medium composed of Ham's F10 + 20% fetal bovine serum (FBS) + 1% penicillin/ streptomycin + 25 ng/ml bFGF (Sigma) + 10 ng/ml IGF-1 (Sigma). bFGF and IGF1 were replaced daily. After 7 days, growth medium was replaced with myogenic differentiation medium composed of Ham's F10 + 2% FBS + 1% Pen-Strep. Cells were cultured in myogenic differentiation medium for 4-5 days, fixed in 4% PFA for 20 min at RT and blocked prior to staining in PBS containing 20% normal goat serum. Cells were stained with primary antibody (monoclonal mouse anti-DESMIN antibody, clone D33, M0760, titer 1:50, Dako, Carpinteria, CA) at 4°C overnight and with secondary antibody (goat anti-mouse Alex Fluor 594 conjugate, Invitrogen, titer 1:200) for one hour at RT. Nuclei were stained with Hoechst (2 μg/ml for 20 minutes at RT). Immunofluorescent labeling was analyzed using an Olympus IX51 microscope at 20X.

Adipogenic Differentiation Assay

Human MFA cell subpopulations were sorted at 4 x 10^3 cells/ well in 96 well plates. Cells were expanded in adipogenic growth medium composed of 60% DMEM low glucose + 40% MCDB201 medium + 2% FBS + 1% Pen-Strep + 1 nM Dexamethasone (Sigma) + 0.1 mM L-Ascorbic Acid 2-Phosphate (Sigma) + ITS mix (1 in 100, Sigma) + Linoleic Acid-Albumin (1 in 100, Sigma) + 25 ng/ml bFGF (Sigma) until cells reached 100% confluence (13-14 days). bFGF was replaced daily. Medium was then replaced with adipogenic induction medium composed of 60% DMEM low glucose + 40% MCDB201 medium + 2% FBS + 1% Pen-Strep + 1 μM Dexamethasone (Sigma) + 5 μg/ml Insulin (Roche, Basel, Switzerland) + 0.5 mM IBMX (Sigma) + 1 nM T3 (Sigma) + 1 μM Roziglitazone (Sigma) for 3 days. After

3 days, medium was replaced with adipogenic differentiation medium consisting of 60% DMEM low glucose + 40% MCDB201 Media + 2% FBS + 1% Pen-Strep, 5 μg/ml Insulin (Sigma) + 1 nM T3 (Sigma) + 1 μM Roziglitazone (Sigma) for 4 days. Cells were fixed with 4% PFA for 20 minutes at RT, stained with Oil Red O (Sigma) for 1 hour at room temperature and then washed with dH_2O several times until the supernatant was clear. Oil Red O staining of lipid droplets within adipocytes was analyzed using an Olympus IX51 inverted microscope at 20X.

Osteogenic Differentiation Assay

Human MFA cell subpopulations were sorted at 4 x 10^3 cells/ well (1 x 10^3 cells/ well in case of adult human MFA cells) in 96 well plates. Cells were expanded in Preadipocyte Medium (PM-1, ZenBio, Research Triangle Park, NC) + 25 ng/ml bFGF (Sigma) until they reached 100% confluence (13-14 days). bFGF (Sigma) was replaced daily. Medium was then replaced with Osteoblast Differentiation Medium (OB-1, ZenBio). Cells were kept in OB-1 for 14 days, fixed in ice cold 70% ethanol for 1 hour at 4°C, stained with 2% Alizarin Red (Sigma), pH 4.2 for 10 minutes at RT and then washed with dH_2O several times until the supernatant was clear. Alizarin red staining was analyzed using an Olympus IX51 microscope at 20X.

Clonal Cell Culture

Fetal hMFA cell subpopulations were sorted at 1 cell/well in 96 well plates coated with 1 μg/ml rat-tail COLLAGEN (Sigma) and 10 μg/ml natural mouse LAMININ (Invitrogen). Cells were cultured in myogenic growth medium. 25 ng/ml bFGF (Sigma) and 10 ng/ml IGF-1 (Sigma) were added daily. After 9-10 days, cell growth was evaluated by standard microscopy using an Olympus IX51 microscope at 20X. The number of wells with visible

cell growth out of all wells that received one cell was determined. Cells were kept in myogenic growth conditions until they reached 100% confluence and then passaged by aspiration of medium and re-plated into 2% Matrigel (BD) coated 96-well plates. Cells were used for either myogenic or osteogenic differentiation assays as outlined above.

Transplantation studies

The tibialis anterior (TA) muscle of 6-8 week-old male and female NSG transplant recipients was conditioned 24 hours prior to transplantation of human cells by injection of 25μl (0.03mg/ml) of Naja mossambica mossambica cardiotoxin (CTX, Sigma). Unfractionated fetal hMFA cells and fetal hMFA subsets were FACS sorted and injected in 25 μl of SM into the conditioned TA muscles of anesthesized recipient mice. 3-8 weeks after transplantation, recipient muscles were harvested, fixed by freezing in Methylbutane (Sigma) and stored at -80°.

Engraftment was evaluated by IF staining for h-SPECTRIN (human species-specific) and DYSTROPHIN. Serial 7 μm sections of recipient TA muscles were obtained using a Cryostat. Tissue was permeabilized by exposure to 0.2% Triton-X for 20 min at room temperature and incubated 1 hour at RT in blocking solution (Papain digested RAM antibodies supplemented with goat Fc antibodies at 5 μg/ml and 5% FBS according to previously published protocols, (Ehrhardt et al., 2007)). Endogenous biotin was blocked using the Avidin/ Biotin blocking kit (Vector). Tissue was stained with primary antibodies against h-SPECTRIN (1:20, mouse anti-human SPECTRIN antibody, clone RBC2/3D5, Leica) and DYSTROPHIN (1:50, rabbit polyclonal anti-DYSTROPHIN antibody, ab15277, Abcam) at 4°C overnight. For h-SPECTRIN staining, tissue was then exposed to a biotinylated goat anti-mouse secondary antibody (1:350, Dako) for one hour at RT and to Alexa-Flour-594-labeled streptavidin (1:200, Invitrogen) for 45 min at room temperature.

For DYSTROPHIN staining, tissue was stained with an anti-rabbit Alexa-Flour-488-labeled secondary antibody (1:200, Invitrogen). Slides were coverslipped using Vectashield mounting media with DAPI to stain nuclei. IF staining was analyzed using an Olympus BX60 upright microscope at 20-40X.

Microarray Analysis

Unfractionated fetal hMFA, $CD34^{-}CD56^{int}ITGA7^{hi}$ and $CD34^{+}$ cells were sorted by FACS from 3-4 biologically independent human fetal skeletal muscle specimens as described above. Total RNA was obtained using TRIzol extraction. RNA quantity and quality was determined by Nanodrop and Agilent 2100 Bioanalyzer evaluation (Harvard Medical School Biopolymers Facility Service). Only samples with a purity of > 99% (Fig S3) and a RIN score > 7 were included in the microarray analysis. RNA was labeled and hybridized to Affymetrix microarrays (Human Genome U133 Plus 2.0). Array quality was confirmed using RMA and Affymetrix command console modules. Microarray data obtained from human MFA cell subsets were deposited in the NCBI database under accession number GSE44227.

Raw data were normalized in batch against an invariant set. Differentially regulated probesets were identified using GenePattern. Hierarchical clustering was performed in GenePattern (Broad Institute). Principal Component analysis (PCA) was performed using 3D-PCA. Row- and column-normalized and $\log_2$-transformed data were used with the default settings of minimum expression value 120 (EV>120 for any dataset) and 20% most variable using the PCA Plot module (GenePattern, Broad Institute). The first three principle components (PC1, PC2 and PC3, respectively) were used as coordinate-axes onto which samples were projected. Pathway analysis was performed within the cluster of genes upregulated in fetal $CD34^{-}CD56^{int}ITGA7^{hi}$ cells versus $CD34^{+}$ cells and vice versa using

Ingenuity. Cell surface location of differentially regulated transcripts was screened using Ingenuity.

PCR

Total RNA was isolated by TRIzol extraction from 2 biologically independent fetal $CD34^{-}CD56^{int}ITGA7^{hi}$ samples, 3 biologically independent fetal $CD34^{+}$ hMFA samples and 4 discrete $CD34^{-}CD56^{int}ITGA7^{hi}$ clones subjected to myogenic and osteogenic differentiation. RNA was reverse transcribed using Superscript III First-Strand Synthesis System for RT-PCR (Invitrogen). qRT-PCR was performed using an AV7900 PCR system (Applied Biosystem) and Taqman Gene Expression Assays (Invitrogen): *PAX7* (Hs00242962_m1), *MYF5* (Hs00929416_g1), *PPARG* (Hs01115513_m1), *FABP4* (Hs01086177_m1), *BGLAP* (Hs01587814_g1), *RUNX2* (Hs00231692_m1), *SP7* (Hs01866874_s1), *DESMIN* (Hs01090875_m1), *MYOD1* (Hs02330075_g1), *GAPDH* (Hs02758991_g1).

Figure S1

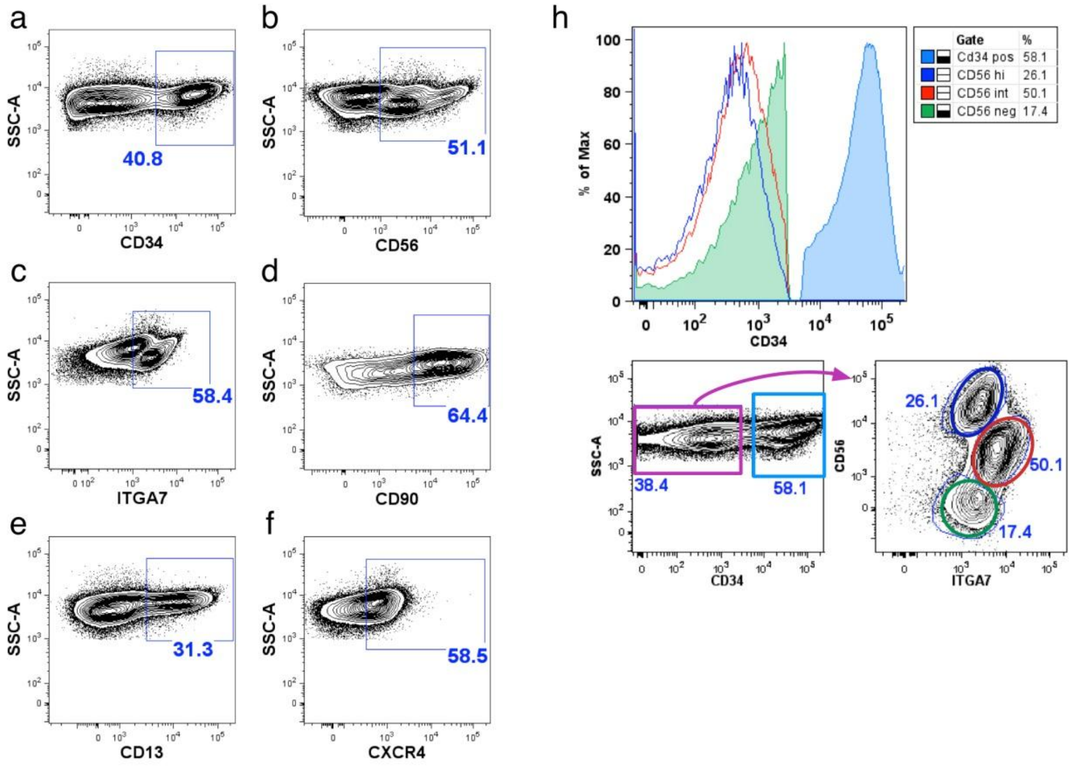

Figure S1. Expression of cell surface antigens by flow cytometry in live (Pi^-Ca^+ or $7AAD^-Ca^+$) human fetal MFA cells. (a) CD34. (b) CD56. (c) ITGA7. (d) CD90. (e) CD13. (f) CXCR4. The percent of cells positive for each marker is indicated on each plot. (h) Back-gating analysis of hMFA subsets revealed that $CD34^{-/low}CD56^{-}ITGA7^{low}$ cells (green) expressed relatively higher levels of CD34 than $CD34^{-}CD56^{int}ITGA7^{hi}$ (red) and $CD34^{-}CD56^{hi}ITGA7^{low}$ (blue).

Figure S2

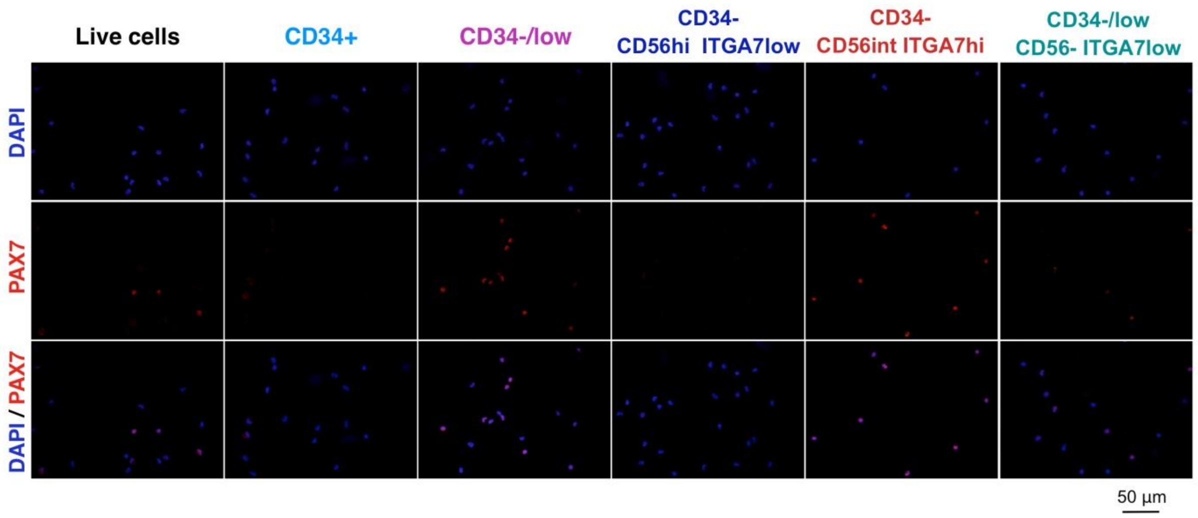

Figure S2. PAX7 expression by immunofluorescence in prospectively isolated fetal hMFA cell subsets.

Figure S3

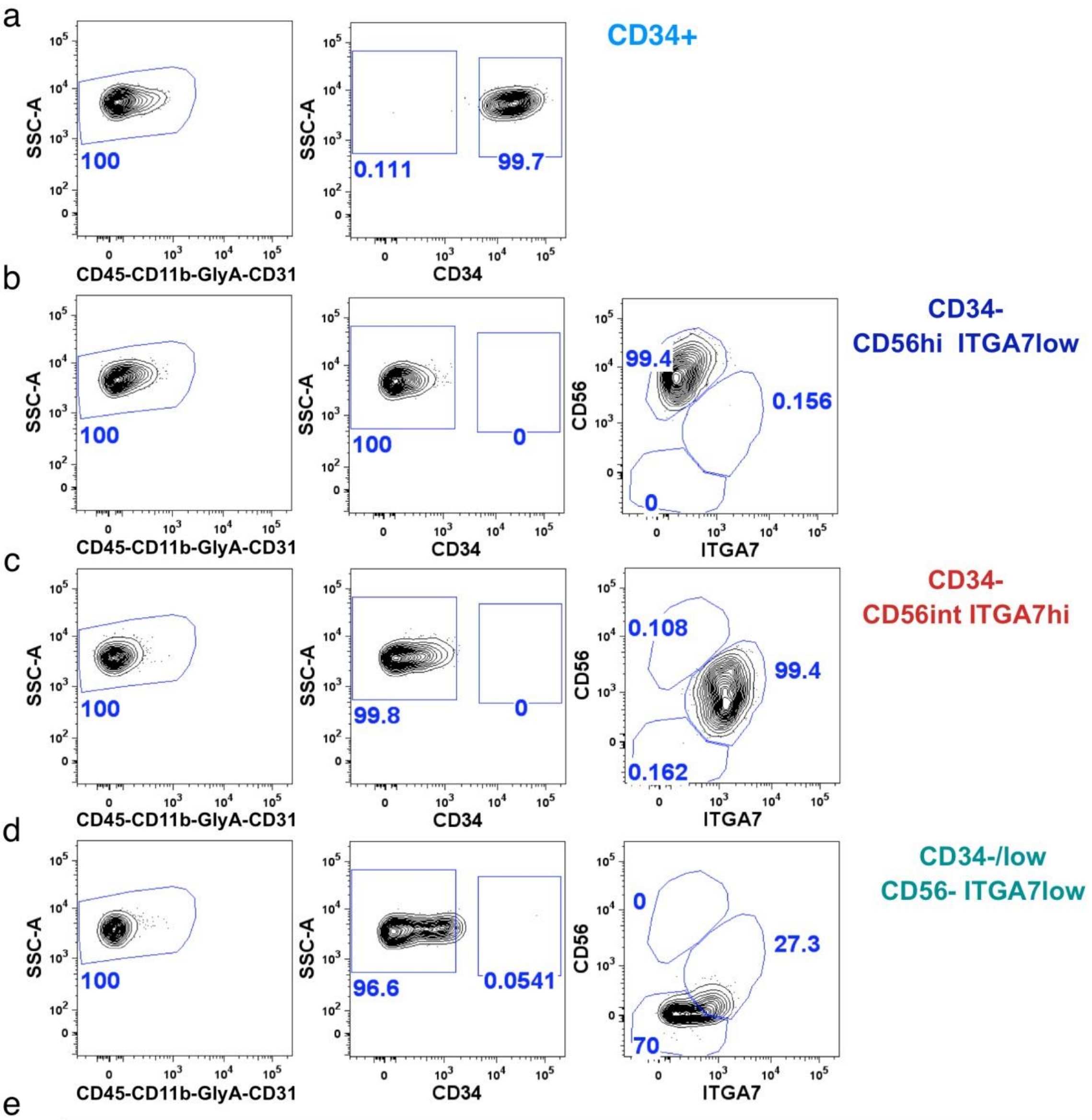

e

Sample ID	CD34+	CD34-<br>CD56hi ITGA7low	CD34-<br>CD56int ITGA7hi	CD34-/low<br>CD56- ITGA7low
312601/8127	99.5	99.6	99.3	72
931501/2867	99.7	99.4	99.4	33.5
641202/8227	99.7	99.4	99.4	70
250805/4114	100	99.6	99.4	96.9

Figure S3. Purity of FACS-sorted fetal hMFA cell subsets. The purity of sorted CD34^{+} cells (a), CD34^{-}CD56^{hi}ITGA7^{low} cells (b), and CD34^{-}CD56^{int}ITGA7^{hi} cells (c) was consistently > 99% (e). The purity of sorted CD34$^{-/low}$ cells was 99.7-99.9% (data not shown). The purity of

sorted CD34$^{-/low}$CD56^{-}ITGA7^{low} cells (d) was highly variable, ranging from 33.5-96.9% (e). All samples were double-sorted, and then re-analyzed to assess purity. Purity was determined as percentage of cells with defined cell surface marker expression profiles out of all viable cells in 4 biologically and technically independent experiments.

Figure S4

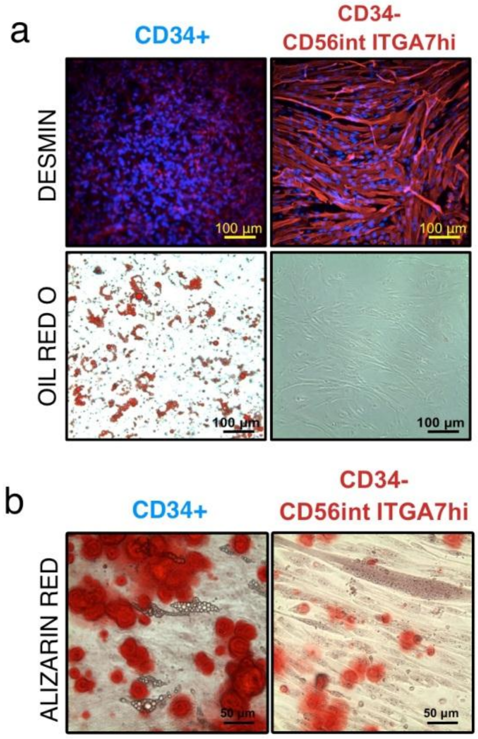

Figure S4. Myogenic differentiation capacity of hMFA cell subsets under adipogenic (a) or osteogenic (b) conditions in vitro. (a) Cells were stained for DESMIN (red, top row), or for lipid (Oil Red O, bottom row). CD34^{-}CD56^{int}ITGA7^{hi} cells maintain their capacity to differentiate into DESMIN+ myotubes under adipogenic conditions. (b) Some CD34^{-}CD56^{int}ITGA7^{hi} cells differentiate into elongated fibers under osteogenic conditions.

Figure S5

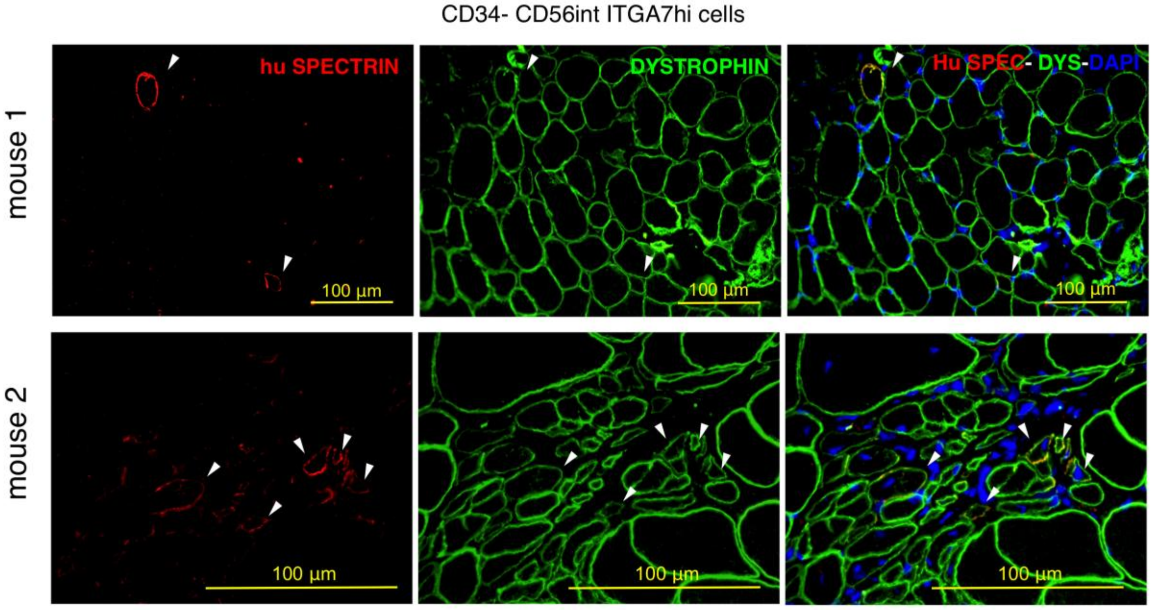

Figure S5. In vivo myogenic engraftment of fetal human CD34$^-$CD56^{int}ITGA7^{hi} hMFA cells. Human CD34$^-$CD56^{int}ITGA7^{hi} hMFA cells engrafted to form myofibers as demonstrated by double staining for h-SPECTRIN (red) and DYSTROPHIN (green). Representative images from 2 mice, engrafted with fetal human CD34$^-$CD56^{int}ITGA7^{hi} hMFA cells, are shown.

Figure S6

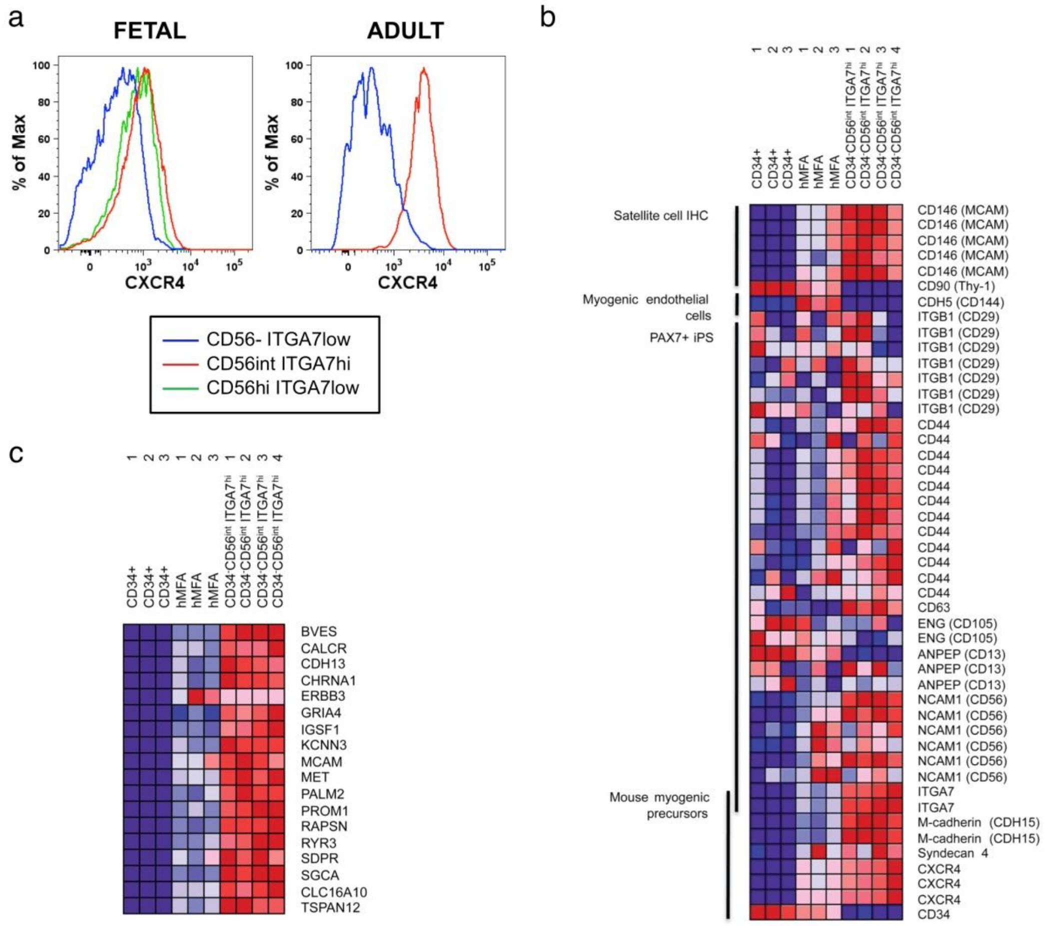

Figure S6. Additional cell surface markers for human myogenic/osteogenic progenitor cells in muscle. (a) Differential expression of CXCR4 in fetal and adult $CD34^{-}CD56^{int}ITGA7^{hi}$ hMFA cells. Adult $CD34^{-}CD56^{int}ITGA7^{hi}$ hMFA cells show higher levels of expression of CXCR4 compared to $CD56^{-}ITGA7^{low}$ cells by flow cytometry, whereas fetal $CD34^{-}CD56^{int}ITGA7^{hi}$ hMFA cells exhibit similar levels of CXCR4 compared to $CD56^{-}ITGA7^{low}$ cells. (b) Differential expression of selected cell surface antigens, previously linked to mouse and/or human myogenic precursor cells (Cerletti et al., 2008; Darabi et al., 2012; Lecourt et al., 2010; Zheng et al., 2007) in fetal hMFA cell subsets (Blue, down-regulated genes; Red, up-regulated genes). Microarray analysis demonstrated increased transcript levels of *CXCR4*,

CD44 and *MCAM* (CD146) in fetal CD34$^-$CD56^{int}ITGA7^{hi} hMFA cells . (c) mRNAs for cell surface markers that are upregulated in fetal CD34$^-$CD56^{int}ITGA7^{hi} hMFA cells compared to CD34$^+$ cells. Within the cluster of genes upregulated in fetal CD34$^-$CD56^{int}ITGA7^{hi} hMFA cells compared to CD34+ cells (>15-fold, p<0.01), those genes corresponding to transcripts localizing to the cell membrane were identified using Ingenuity (Blue, down-regulated genes; Red, up-regulated genes).

Figure S7

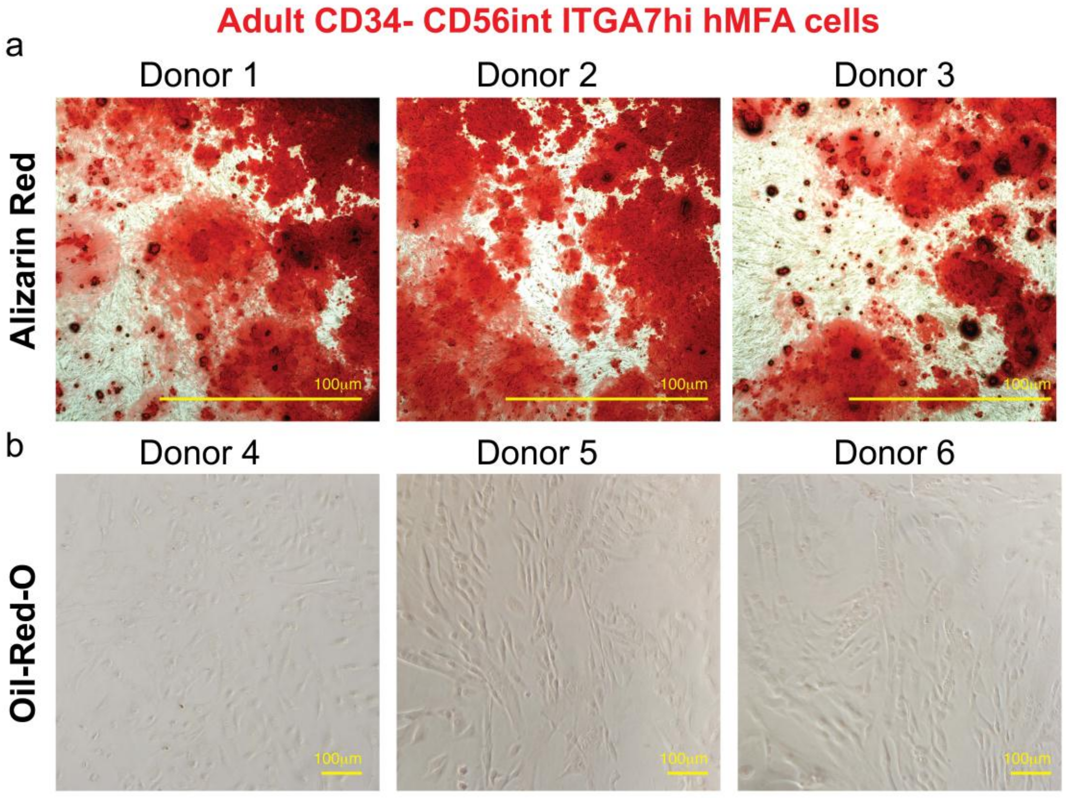

Figure S7. Osteogenic and adipogenic differentiation capacity of adult hMFA subsets. (a) CD34$^-$CD56^{int}ITGA7^{hi} adult hMFA cells, obtained from three different adult donors (BI111212, BI111912, BI110512), formed Alizarin Red positive calcium deposits. (b) CD34$^-$CD56^{int}ITGA7^{hi} adult hMFA cells, obtained from three different adult donors, did not

differentiate into ORO-positive adipocytes. Thus, osteogenic differentiation potential and lack of adipogenic capacity of $CD34^-CD56^{int}ITGA7^{hi}$ hMFA cells is similar in adult and fetal skeletal muscle.

Tables

Table S1. List of human fetal and adult skeletal muscle samples used in this study. The proportions of cells marked by specific combinations of cell surface markers are expressed as percentage of Pi^-7AAD^+ or Pi^-Ca^+ (live) fetal hMFA cells.

Table S2. Genes upregulated in fetal $CD34^-CD56^{int}ITGA7^{hi}$ versus $CD34^+$ cells (> 5-fold, p< 0.01).

Table S3. Top functions among genes upregulated in fetal $CD34^-CD56^{int}ITGA7^{hi}$ versus $CD34^+$ cells as per Ingenuity pathway analysis.

Table S4. Genes upregulated in fetal $CD34^+$ versus $CD34^-CD56^{int}ITGA7^{hi}$ cells (> 5-fold, p< 0.01).

Table S5. Top functions among genes upregulated in fetal $CD34^+$ versus $CD34^-CD56^{int}ITGA7^{hi}$ cells as per Ingenuity pathway analysis.

Table S6. Cell membrane molecules differentially regulated in fetal $CD34^-CD56^{int}ITGA7^{hi}$ compared to $CD34^+$ cells (fold-change >15, p<0.01). Within the cluster of genes upregulated

in fetal $CD34^-CD56^{int}ITGA7^{hi}$ compared to $CD34^+$ cells (>15-fold, p<0.01), those genes corresponding to transcripts localizing to the cell membrane were identified using Ingenuity.

Table S7. List of antibodies used for flow cytometry analysis and prospective FACS sorting of discrete hMFA subsets.
